# Long-Term Clinical and Immunological Profile of Kidney Transplant Patients Given Mesenchymal Stromal Cell Immunotherapy

**DOI:** 10.3389/fimmu.2018.01359

**Published:** 2018-06-14

**Authors:** Norberto Perico, Federica Casiraghi, Marta Todeschini, Monica Cortinovis, Eliana Gotti, Valentina Portalupi, Marilena Mister, Flavio Gaspari, Alessandro Villa, Sonia Fiori, Martino Introna, Elena Longhi, Giuseppe Remuzzi

**Affiliations:** ^1^IRCCS – Istituto di Ricerche Farmacologiche Mario Negri, Bergamo, Italy; ^2^Unit of Nephrology and Dialysis, Azienda Socio Sanitaria Territoriale Papa Giovanni XXIII, Bergamo, Italy; ^3^G. Lanzani Laboratory of Cell Therapy, Azienda Socio Sanitaria Territoriale Papa Giovanni XXIII, Bergamo, Italy; ^4^Laboratory of Transplant Immunology, UOC Coordinamento Trapianti IRCCS Fondazione Ca’ Granda – Ospedale Maggiore Policlinico di Milano, Milan, Italy; ^5^L. Sacco Department of Biomedical and Clinical Sciences, University of Milan, Milan, Italy

**Keywords:** mesenchymal stromal cells, kidney transplantation, tolerance, memory CD8^+^ T cells, regulatory T cells, B cells

## Abstract

We report here the long-term clinical and immunological results of four living-donor kidney transplant patients given autologous bone marrow-derived mesenchymal stromal cells (MSCs) as part of a phase 1 study focused on the safety and feasibility of this cell therapy. According to study protocols implemented over time, based on initial early safety findings, the patients were given MSC at day 7 posttransplant (*n* = 2) or at day −1 pretransplant (*n* = 2) and received induction therapy with basiliximab and low-dose rabbit anti-thymocyte globulin (RATG) or RATG alone, and were maintained on low-dose ciclosporin (CsA)/mycophenolate mofetil (MMF). All MSC-treated patients had stable graft function during the 5- to 7-year follow-up, without increased susceptibility to infections or neoplasm. In three MSC recipients, but not historical control patients, circulating memory CD8^+^ T cell percentages remained lower than basal, coupled with persistent reduction of *ex vivo* donor-specific cytotoxicity. Two patients showed a long-lasting increase in the regulatory T cell/memory CD8^+^ T cell ratio, paralleled by high circulating levels of naïve and transitional B cells. In one of these two patients, CsA was successfully discontinued, and currently the low-dose MMF monotherapy is on the tapering phase. The study shows that MSC therapy is safe in the long term and could promote a pro-tolerogenic environment in selected patients. Extensive immunomonitoring of MSC-treated kidney transplant recipients could help selection of patients for safe withdrawal of maintenance immunosuppressive drugs (NCT00752479 and NCT02012153).

## Introduction

Kidney transplantation has become the treatment of choice for patients with end-stage renal disease (ESRD). Calcineurin inhibitors and other powerful immunosuppressive agents have led to significant improvements in short-term allograft survival over the last two decades but have had only marginal effects on preventing chronic rejection and prolonging long-term graft longevity (1). Furthermore, life-long non-specific immunosuppression increases the rates of malignancy, opportunistic infections and, through drug-specific side effects, the risk of cardiovascular and metabolic diseases (2, 3). Therefore, the transplant community’s attention has now shifted away from harmful immunosuppressive regimens toward identifying strategies to induce specific allograft tolerance, avoiding or limiting the need for conventional anti-rejection medications (4). In this perspective, three medical centers are actively pursuing tolerance induction in recipients of living-donor kidney transplants by infusing donor hematopoietic stem cells to establish various levels of chimerism (5–8). The protocols adopted, with differing conditioning regimens, donor cell products and immunosuppressive treatment, enabled successful withdrawal of immunosuppression in a subset of patients, demonstrating the feasibility and efficacy of cell-based therapies in inducing tolerance in kidney transplant recipients (5–8). Unfortunately, the risks of serious infections, graft-vs-host disease, and ultimately death, inherently associated with the procedures, clearly outweigh any potential benefits of tolerance, making these protocols too risky to justify routine use in patients without malignancies. Thus, alternative immunomodulatory cell populations not requiring peri-transplant conditioning regimens are currently being investigated for use as immunotherapy in solid organ transplantation, including tolerogenic dendritic cells, regulatory macrophages, regulatory T cells (Tregs), and mesenchymal stromal cells (MSCs) (9, 10).

In 2011, we first reported the early results of a pilot safety and feasibility study based on infusion of autologous bone marrow-derived MSC in two recipients of kidney transplant from a living-related donor, 7 days after transplantation (11). Unexpectedly, transient renal insufficiency (engraftment syndrome) was observed in both patients 7–10 days after MSC infusion. Thus, based on findings in a kidney transplant model, the clinical protocol was amended to plan MSC infusion the day before transplantation, and two additional living-related donor kidney transplant patients given autologous MSC no longer experienced engraftment syndrome (12). This pre-specified result-driven approach has shown that this cell therapy was clinically feasible and safe, provided MSCs were administered before transplantation (11, 12). Moreover, MSC infusion promoted the development of a pro-tolerogenic milieu, characterized by an increase in the ratio between Tregs and memory CD8^+^ T cells in the peripheral blood as well as a profound and persistent reduction in *ex vivo* anti-donor CD8^+^ T cell cytolytic function until 12-month follow-up in all of these four patients (11, 12). More recently, other groups have also described the short-term safety and feasibility of MSC-based therapy in living-donor kidney transplant recipients (13–17). Furthermore, there are some indications of the early MSC efficacy in allowing safely adoption of lower immunosuppressive drug regimens than currently used (13, 15, 17). However, so far no data have been reported on the long-term impact of this novel immunomodulatory approach to solid organ transplantation.

Here, we report on longer 5- to 7-year follow-up on our initial cohort of MSC-treated patients, focusing on their long-term clinical course for graft outcomes and possible adverse events, and assessing whether the pro-tolerogenic milieu is sustained and long-lasting by sequential monitoring of Treg and memory CD8^+^ T cell profile and donor-specific host immune response. Moreover, we describe early results of a new patient who received pretransplant infusion of MSC, to confirm the initial biological/mechanistic effects of this cell treatment documented in the initial cohort.

## Materials and Methods

### Study Protocols

All treatment protocols were approved by the Istituto Superiore di Sanità [ISS, Rome, authorization number no. 45253(06)-PRE.21-882 and no. 28689(13)321-1223] and by Agenzia Italiana del Farmaco (AIFA) on October 10, 2007 and September 30, 2013, respectively, and by the Institutional Review Board of the Ospedali Riuniti/Azienda Ospedaliera Papa Giovanni XXIII of Bergamo (authorization no. 352, March 18, 2008 and no. 110/13, November 6, 2013). The study is registered with ClinicalTrials.gov (NCT00752479 and NCT02012153). Written informed consent was obtained from all recipients and living donors in accordance with the Declaration of Helsinki.

**Figure 1:**
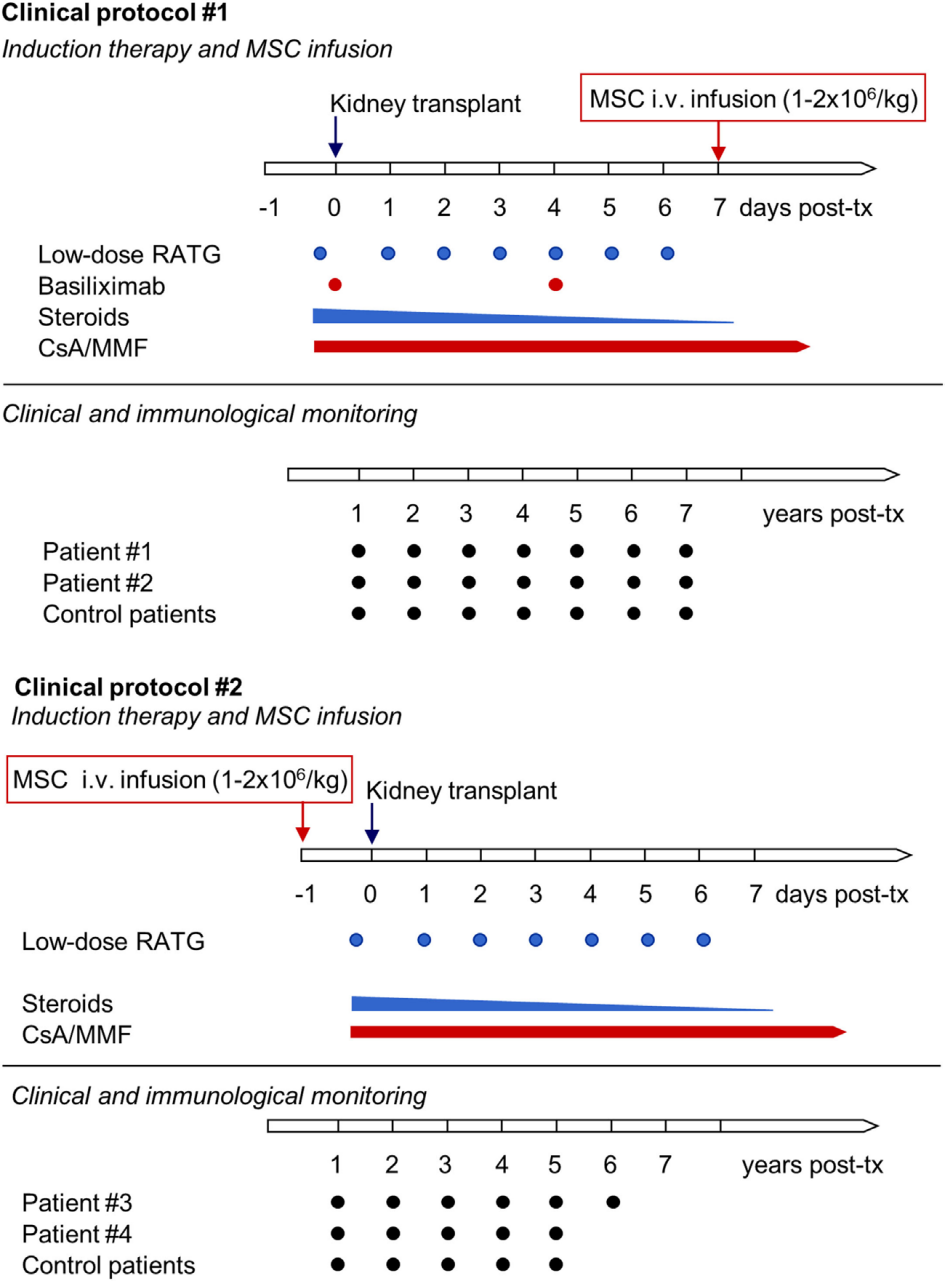
Clinical protocols of MSC-treated kidney transplant recipients implemented at our center. The first two living-related donor kidney transplant recipients adopted the initial clinical protocol (Clinical protocol #1), where autologous bone marrow-derived MSC were infused 7 days after transplantation and induction therapy comprised basiliximab and low-dose RATG. Since in both patients transient acute renal insufficiency occurred due to engraftment syndrome, the protocol was revised (Clinical protocol #2), and two subsequent patients received pretransplant MSC infusion and induction therapy with low-dose RATG alone. Abbreviations: CsA, ciclosporin; MMF, mycophenolate mofetil; MSC, mesenchymal stromal cell; RATG, rabbit anti-thymocyte globulin.

Patients with ESRD were enrolled in a phase 1, single-center, open-label pilot study conducted at the Ospedale Papa Giovanni XXIII, Bergamo, Italy, that aimed primarily to characterize the safety and feasibility of peri-transplant infusion of *ex vivo* expanded, autologous bone marrow-derived MSC in living-related donor kidney transplant recipients. Patients #1 and #2 adopted the initial study protocol (Protocol 1, see below), with MSC given intravenously 7 days posttransplantation (Figure 1) (11). Since transient acute renal insufficiency due to engraftment syndrome occurred after cell infusion, the protocol was revised (Protocol 2, see below) in the next patients, #3 and #4, who received pretransplant (day −1) MSC infusion (Figure 1) (12). Moreover, patients #1 and #2 received induction therapy with basiliximab and low-dose rabbit anti-thymocyte globulin (RATG), whereas patients #3 and #4 were given RATG alone to avoid the possible negative impact of basiliximab on Treg development and function (11, 12). As controls for patients #1 and #2, six kidney transplant recipients from living-related (*n* = 3) and deceased (*n* = 3) donors given basiliximab/low-dose RATG as induction therapy were considered (11). For patients #3 and #4, an additional control group (*n* = 6) of historical deceased-donor kidney transplant recipients receiving the same low-dose RATG induction therapy alone was studied, since in our living-donor transplant program only dual induction therapy with basiliximab and low-dose RATG is adopted. In all MSC-treated and control patients, maintenance immunosuppressive therapy comprised low-dose ciclosporin (CsA) and low-dose mycophenolate mofetil (MMF) (Figure 1).

MSC-treated patients and the two groups of control kidney transplant recipients were comparable for gender, cause of renal failure, and donor age (Table 1).

**Table 1:** Demographic characteristics and clinical course in MSC-treated patients and in control groups of kidney transplant recipients.

	Age at Tx (years)/gender (M:F)	Donor age (years)	Cause of renal failure	HLA mismatch median (range)	Post-Tx follow-up (months)	Serum creatinine at month 6 post-Tx (mg/dL)	Serum creatinine at month 12 post-Tx (mg/dL)	Serum creatinine at last follow-up (mg/dL)	Measured GFR at month 6 post-Tx (mL/min/1.73 m^2^)	Measured GFR at month 12 post-Tx (mL/min/1.73 m^2^)	Measured GFR at month 60 post-Tx (mL/min/1.73 m^2^)	Measured GFR at last follow-up (mL/min/1.73 m^2^)	Proteinuria at month 60 post-Tx (g/24 h)	Proteinuria at last follow-up (g/24 h)
**MSC-treated patients**														

MSC #1	22/M	43	Unknown	2	84	1.83	1.93	2.27	49.59	48.41	47.30	46.61	0.65	0.91
MSC #2	34/M	64	IgA nephropathy	2	84	2.38	2.24	2.21	38.90	38.52	41.25	30.73	0.26	1.24
MSC #3	37/M	65	IgA nephropathy	2	72	1.35	1.23	0.97	62.79	59.16	69.14	76.68	0.07	0.15
MSC #4	34/M	61	Medullary sponge disease	3	60	1.37	1.25	1.49	51.06	51.04	48.36	48.36	0.06	0.06

**Control patients**														

Bas/low-dose RATG control group	42 ± 15/4:2	44 ± 4	IgA nephropathy (*n* = 3), ADPKD (*n* = 1), lupus nephritis (*n* = 1), and unknown (*n* = 1)	2 (0–4)	84	1.47 ± 0.40	1.54 ± 0.34	1.77 ± 0.22	44.83 ± 7.44	49.41 ± 6.79	48.60 ± 5.36	40.77 ± 5.22	0.42 ± 0.29	0.54 ± 0.31

Low-dose RATG control group	56 ± 9/5:1	51 ± 5	IgA nephropathy (*n* = 1), ADPKD (*n* = 2), unknown (*n* = 2), and nephroangiosclerosis (*n* = 1)	4 (3–5)	60	1.58 ± 0.28	1.45 ± 0.31	1.94 ± 0.40	44.71 ± 5.09	46.01 ± 6.47	42.18 ± 5.44	42.18 ± 5.44	0.52 ± 0.39	0.52 ± 0.39

*Data from control groups are mean ± SEM*.

*ADPKD, autosomal dominant polycystic kidney disease; Bas, basiliximab; GFR, glomerular filtration rate; IgA, immunoglobulin A; RATG, rabbit anti-thymocyte globulin; Tx, transplantation; HLA, human leukocyte antigen; MSC, mesenchymal stromal cell*.

### Protocol 1

Four months before transplantation patients underwent sterna bone marrow aspiration under local anesthesia. MSCs were isolated and *ex vivo* expanded according to Good-Manufacturing Practice procedures (18, 19). On day 7 after kidney transplant, autologous MSCs were administered intravenously (1.7 × 10^6^ and 2.0 × 10^6^ cells/kg body weight, respectively) after premedication with chlorphenamine and acetaminophen. Patients received induction regimen with basiliximab (20 mg intravenously pretransplant and on day 4 posttransplant) and low-dose RATG infusion (thymoglobulin, 0.5 mg/kg, daily from day 0 to day 6 posttransplant) as per center practice (20). Maintenance immunosuppression was with CsA (target trough blood levels of 300–400 ng/mL up to day 7 postsurgery, and 100–150 ng/mL at month 5 posttransplantation), MMF, and steroids. Five hundred milligrams of methylprednisolone were administered before the first RATG infusion to minimize the possible cytokine release reaction related to the antibodies, and continued for two more days posttransplant (250 and 125 mg, respectively). Subsequently, oral prednisone (75 mg) was administered, which was progressively tapered and discontinued after day 7 postsurgery.

### Protocol 2

Four to six months before transplantation patients underwent right posterior superior iliac crest aspiration under local anesthesia. MSCs were isolated and *ex vivo* expanded according to Good-Manufacturing Practice procedures. The day before kidney transplantation (day −1) autologous MSCs were administered intravenously (2.0 × 10^6^ cells/kg body weight) after premedication with chlorphenamine and acetaminophen. Patients received induction therapy with low-dose RATG infusion (0.5 mg/kg, daily from day 0 to day 6 posttransplant). Maintenance immunosuppression was with CsA (target trough blood levels of 300–400 ng/mL up to day 7 postsurgery, and 100–150 ng/mL at month 5 posttransplantation), MMF and steroids. Five hundred milligrams of methylprednisolone were administered before the first RATG infusion to minimize the possible cytokine release reaction related to the antibodies, and continued for two more days posttransplant (250 and 125 mg, respectively). Subsequently, oral prednisone (75 mg) was administered, which was progressively tapered and discontinued after day 7 postsurgery.

### Patient Follow-Up

In all MSC-treated patients clinical and hematochemical parameters, acute graft rejection episodes and incidence and severity of adverse events have been prospectively evaluated up to their last clinical follow-up, spanning from 5 to 7 years after transplantation. Similar parameters were retrospectively evaluated in control patients during the same follow-up period after kidney transplantation. Both MSC-treated and control transplant recipients had periodically monitoring of renal graft function as glomerular filtration rate (GFR), measured by the plasma clearance of iohexol (21, 22), every 6 months after kidney transplantation. At the same time points, 24 h urinary samples were collected to evaluate urinary protein excretion. Immunophenotyping of peripheral blood lymphocyte subpopulations, *ex vivo* functional immunological assays to assess donor-specific hyporesponsiveness and monitoring of donor-specific anti-human leukocyte antigen (HLA) antibodies were sequentially evaluated in MSC-treated patients and compared with those in controls. Donor cells for functional immunological studies were available for six control patients given basiliximab/low-dose RATG, but for only one receiving low-dose RATG alone.

### MSC Isolation and Expansion

Mesenchymal stromal cells were processed and cultured according to Good-Manufacturing-Practice procedures (Centre of Cellular Therapy “G. Lanzani” Azienda Socio Sanitaria Territoriale Papa Giovanni XXIII, Bergamo, Italy, authorization no. aM.-189/2008 AIFA) (18, 19). Cells were classified as MSC based on their ability to differentiate into bone, fat and cartilage, and by flow cytometric analysis (positive for CD44, CD29, CD73, HLA-ABC, CD90, and CD105, but negative for CD14, CD34, CD45, and HLA-DR) responding to defined criteria for MSC established by the International Society for Cellular Therapy (23). The final product was characterized with respect to viability, purity, and therapeutic potential.

### Immunophenotyping of Peripheral Blood Lymphocytes and *Ex Vivo* Functional Immunological Assays

Peripheral blood mononuclear cells (PBMCs) were collected pretransplant, at 12 months posttransplant and at yearly intervals thereafter. PBMCs were stained with fluorochrome-conjugated murine monoclonal antibodies against human CD3 (clone HIT3a), CD4 (clone RPA-T4), CD8 (clone RPA-T8), CD45RA (clone HIT100), CD45RO (clone UCHL1), CD25 (clone M-A251), CD127 (clone HIL-7R-M21), CD19 (clone HIB19), CD27 (clone M-T271), CD38 (clone HIT2), IgD (clone IA6-2), CD24 (clone ML5), and FoxP3 (clone 236A/E7) (all from BD Bioscience). Multicolor flow cytometry was used to identify T- and B-cell subsets with standard technique and equipment (FACSAria, BD Bioscience) (24, 25). At the same time points, alloimmune response against donor and third-party antigens was assessed by IFNγ ELISpot assay and cell-mediated lympholysis (26).

For NK and monocyte subset analysis, 10^6^ PBMC, resuspended in 50 µL Brilliant Buffer (BD Bioscience), were labeled with a mix of antibodies for 30 min. The mix antibodies contained BUV395 mouse anti-human CD45 (clone HI30), BV510 mouse anti-human CD3 (clone HIT3a), APC-R700 mouse anti-human CD27 (clone M-T271), BB700 mouse anti-human CD16 (clone B73.1), BV650 mouse anti-human CD56 (clone B159), BV711 mouse anti-human CD11b (clone D12), BUV737 mouse anti-human CD14 (clone M5E2), PE-CF594 mouse anti-human CD64 (clone 10.1), BV605 mouse anti-human HLA-DR (clone G46-6), and PE-Cy5 mouse anti-human CD33 (clone WM-53), all antibodies were purchased from BD Bioscience. At the end of incubation, PBMCs were washed with Stain Buffer (BD Bioscience). Cells were suspended in Stain buffer, and 10 min prior the analysis, Cell Viability Solution (BD) was added to exclude death cells. Cells were acquired by FACS Fortessa X-20 (BD Bioscience) and analyzed with FlowJo software.

### Detection of Donor-Specific Anti-HLA Antibodies

Serum samples collected before surgery and at yearly intervals after transplantation from MSC recipients and control patients given basiliximab/low-dose RATG induction therapy were tested for class I and class II anti-HLA antibodies using LABScreen Mixed kit (One Lambda, Canoga Park, CA, USA) (27). Positive tests were quantified by Single-Antigen Beads (One Lambda). A mean fluorescence intensity (MFI) threshold of 2,000 was used to identify a donor-specific anti-HLA antibody (DSA). The tests were carried out according to the manufacturer’s instructions and the analysis was performed with One Lambda software (HLA Visual Version 2.2).

### Statistics

Individual GFR slopes (GFR changes over time) were calculated by linear regression analysis. Difference in GFR slopes between MSC recipients and control groups was assessed by Mann*–*Whitney *U* test. Variations over time in counts and percentages of T- and B-cell subsets, frequency of IFNγ-producing T cells and cell-mediated cytolysis in either control group not given MSCs were assessed by repeated-measures ANOVA, whereas differences between the two control groups were evaluated by ANOVA. All calculations were made using MedCalc 12.3.0.0 (MedCalc Software, Mariakerke, Belgium). *P* value below 0.05 was considered as statistically significant.

## Results

### Patient Demographics and Long-Term Clinical Follow-Up

Table 1 shows demographics and follow-up of MSC-treated patients and controls. Both patients who received MSC infusion at day 7 after transplantation (#1 and #2) developed transient renal dysfunction shortly after cell administration. A graft biopsy few days after MSC infusion in patient #2 excluded acute graft rejection, but showed focal inflammatory infiltrate, mostly granulocytes (11). Following corticosteroid administration, graft function improved and the serum creatinine level stabilized throughout the entire follow-up (7 years) in both patients (patient #1, serum creatinine: 1.83–2.27 mg/dL; patient #2, serum creatinine: 2.38–2.21 mg/dL) (Table 1; Figure S1 in Supplementary Material). At month 77 posttransplantation, proteinuria became detectable in patient #2 and a renal biopsy revealed recurrence of immunoglobulin A nephropathy (Figure S1 in Supplementary Material). In patient #3, in whom pretransplant infusion of MSC proceeded without complications, renal graft function remained normal over the entire 6-year follow-up (serum creatinine: 1.35–0.97 mg/dL) (Table 1; Figure S1 in Supplementary Material). At variance, patient #4, who also received MSC-pretransplant, experienced an acute cellular rejection episode 2 weeks after kidney transplantation. After methylprednisolone pulses, renal function recovered to normal values within 10 days and remained stable until the last available evaluation at 5 years posttransplantation (serum creatinine: 1.37–1.49 mg/dL) (Table 1; Figure S1 in Supplementary Material).

**Figure 2:**
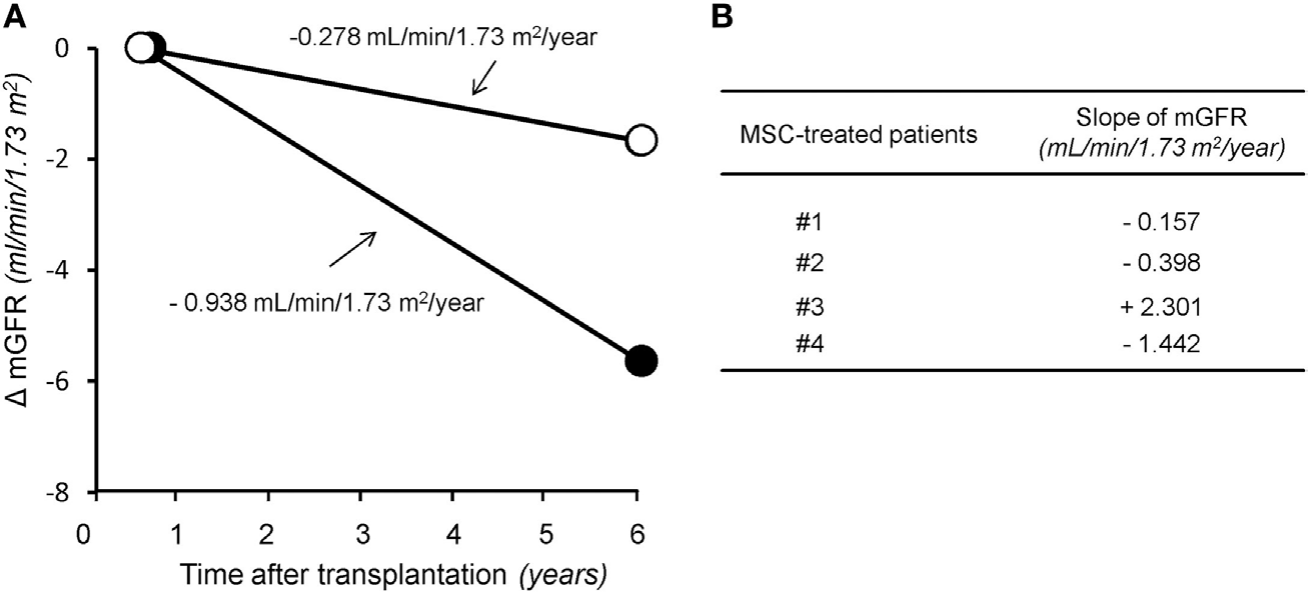
The rate of glomerular filtration rate (GFR) decline in the long term after transplantation. Panel **(A)** represents median slope of GFR in the first four patients given mesenchymal stromal cells (MSC) along with their respective follow-up (open circles) and in control kidney transplant recipients given either basiliximab/low-dose RATG or low-dose rabbit anti-thymocyte globulin (RATG) alone (closed circles) over 7 and 5 years posttransplantation, respectively. Measured GFR decreased to a significantly lower rate in the first four MSC-treated patients compared with living-related donor kidney transplant recipients in the basiliximab/low-dose RATG control group (*n* = 3) (−0.278 vs −1.834 mL/min/1.73 m^2^/year, respectively, *P* < 0.001). Panel **(B)** shows individual slopes of measured GFR in the first four patients given MSC along their respective follow-up.

In the control group given induction therapy with basiliximab/low-dose RATG, serum creatinine levels averaged 1.77 ± 0.22 mg/dL 7 years after transplantation (Table 1). Similarly, in the other control group receiving induction therapy with low-dose RATG alone, mean serum creatinine concentrations were 1.94 ± 0.40 mg/dL at year 5 after transplantation, the average time of follow-up for patients #3 and #4 (Table 1). During the observation periods, one patient in both control groups developed acute cellular rejection that resolved with intravenous methylprednisolone pulses. Interestingly, the median rate of measured GFR decline tended to be numerically slower in the first four MSC recipients throughout their respective follow-up than in control patients given basiliximab/low-dose RATG or low-dose RATG alone (−0.278 vs −0.938 mL/min/1.73 m^2^/year, respectively, *P* = NS) (Figures 2A,B). Similarly, measured GFR decreased to a significantly lower rate in the first four MSC-treated patients compared with living-related donor kidney transplant recipients in the basiliximab/low-dose RATG control group (*n* = 3) (−0.278 vs −1.834 mL/min/1.73 m^2^/year, respectively, *P* < 0.001).

### Safety

Very few serious adverse events were reported in MSC-treated patients, and none was deemed to be related to cell infusion. Table 2 focuses on infectious and neoplastic complications. In the cohort of MSC recipients, a total of seven infectious complications were reported during follow-up. Hospitalization was required only for patient #1 at month 23 after transplantation due to an *Escherichia coli* septic episode originating from the urinary tract (Figure S1 in Supplementary Material). Overall, 20 infectious episodes were recorded in the control group during the long-term observation period, but hospital admission was required for only two patients for cytomegalovirus infection and labyrinthitis at months 2 and 62 posttransplant, respectively (Table 2). One other patient’s initial postsurgery hospitalization was prolonged due to bronchopneumonia (Table 2).

**Table 2:** Infectious and neoplastic adverse events.

	MSC group (*n* = 4)	Basiliximab/low-dose RATG control group (*n* = 6)	Low-dose RATG control group (*n* = 6)
**Infections**			
*Viral*
VZV	0	1	0
HBV	0	1	0
CMV	1	4 (1)	1 (1)
*Bacterial*			
*Escherichia coli* infection	1 (1)	0	0
*Other*
Urinary tract infection	1	4	4
Bronchopneumonia	0	0	1 (1)
Bronchitis	3	0	1
Gastroenteritis	1	0	0
Labyrinthitis	0	1 (1)	0
Tonsillitis	0	2	0
Total	7	13	7
**Neoplasm**			
Renal hemangiomas	1 (1)	0	0
Uterine cervical neoplasm	0	1	0
Total	1	1	0

*CMV, cytomegalovirus; HBV, hepatitis B virus; MSC, mesenchymal stromal cell; VZV, varicella-zoster virus; RATG, rabbit anti-thymocyte globulin*.

*Serious adverse events are indicated in brackets*.

With regard to tumor development, dual benign renal hemangiomas were identified in the right native kidney of patient #2 2 months after transplantation, and dual nephrectomy of the native kidneys was performed (11). A control patient developed squamous papillomas of the uterine cervix 71 months after transplantation and was successfully treated with loop electrosurgical excision procedure (Table 2).

### Immunophenotyping of Peripheral Memory T Cells and Treg

**Figure 3:**
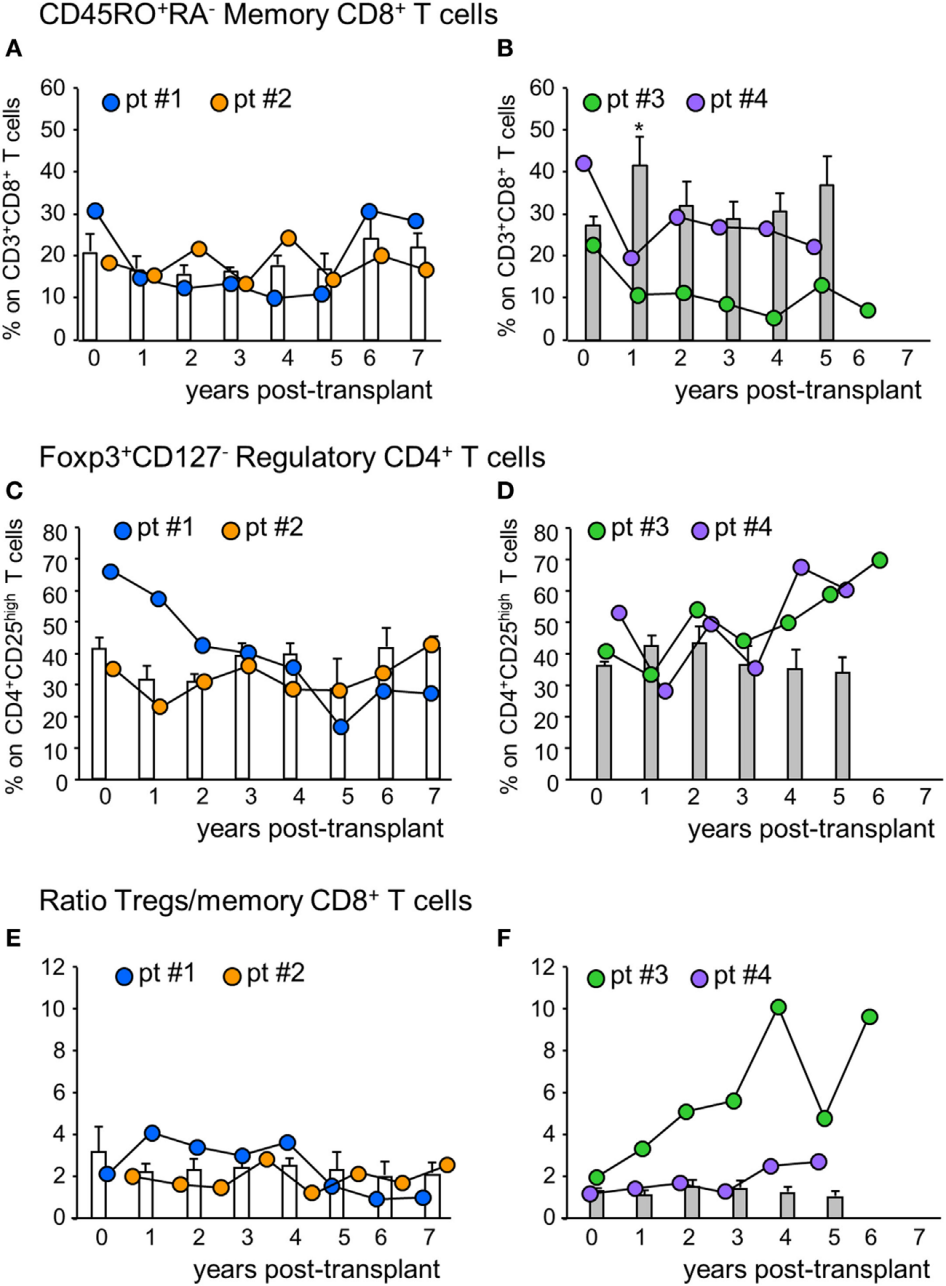
Profile of circulating memory CD8^+^ T cells and regulatory T cells. Percentages of CD45RO^+^RA^−^ memory CD8^+^ T cells (on CD3^+^CD8^+^ T cells) in mesenchymal stromal cell (MSC)-treated patients #1 (blue circles), #2 (orange circles) and in control kidney transplant recipients given basiliximab/low-dose rabbit anti-thymocyte globulin (RATG) induction therapy but not MSC (white histograms) during the follow-up **(A)** and in MSC-treated patients #3 (green circles) and # 4 (violet circles) and in control kidney transplant recipients given induction therapy with low-dose RATG alone but not MSC (gray histograms) during the follow-up **(B)**. Panels **(C,D)** represent profile of percentages of regulatory T cells (Tregs) (Foxp3^+^CD127^−^ on CD4^+^CD25^high^ T cells) during the follow-up in MSC-treated patients #1 and #2 and in control patients given basiliximab/low-dose RATG (white histograms) and in MSC-patients #3 and #4 and in control patients given induction therapy with low-dose RATG alone (gray histograms), respectively. Ratio of Treg/memory CD8^+^ T cell percentages in MSC-treated patients and in control groups are shown in panels **(E,F)**. Data from controls are mean ± SEM, *P < 0.05 vs pretransplant values.

The percentage of circulating memory CD45RO^+^RA^−^CD8^+^ T cells in patient #1 was reduced by 50% at 1-year posttransplant compared with basal value and did not change further until 5 years posttransplant, then returned to pretransplant levels (Figure 3A). In patient #2, conversely, the percentage of memory CD8^+^ T cells did not undergo relevant variations during follow-up. In control patients given basiliximab/low-dose RATG induction therapy, the percentage of memory CD8^+^ T cells remained comparable to pretransplant values during the 7-year observation period (Figure 3A). In MSC recipients #3 and #4, too, the percentage of memory CD8^+^ T cells was reduced by 50% at 1-year posttransplant, compared with pretransplant levels (Figure 3B). While in patient #4 memory CD8^+^ T cell percentage remained lower than basal values until 5-year posttransplant, in patient # 3 the percentage of these cells in the circulation was very low during the entire follow-up period (Figure 3B). At variance, in control patients given induction therapy with low-dose RATG alone, the percentage of memory CD8^+^ T cells significantly increased at 1-year posttransplant compared with pretransplant values, returning to basal levels thereafter (Figure 3B). FACS gating strategy and representative dot plots of memory CD45RO^+^RA^−^CD8^+^ T cells are shown in Figure S2 in Supplementary Material. A very similar trend was found when considering absolute counts of circulating memory CD8^+^ T cells in MSC-treated and control patients (Figure S3 in Supplementary Material).

In the peripheral blood, the percentage of Treg (defined as percentage of Foxp3^+^CD127^−^ on CD4^+^CD25^high^ T cells, see Figure S2 in Supplementary Material) in patients #1 and #2 decreased slightly at 1-year posttransplant compared with the pretransplant value, then further declined progressively in patient #1, while it remained stable in patient #2 (Figure 3C) up to 7 years after transplantation. No significant changes in Treg percentage were documented in control patients given basiliximab/low-dose RATG during follow-up (Figure 3C). Interestingly, in patients #3 and #4, the Treg percentage remained similar to the pretransplant value during the first 3 years posttransplant, with a trend to further increase thereafter, particularly in patient #3 (Figure 3D). This was at variance with control patients given induction therapy with low-dose RATG alone, where the Treg percentage approximated a mean value of 34–45% throughout the entire follow-up (Figure 3D).

When we considered the ratio of Treg/memory CD8^+^ T cells, an increase was observed in patient #1 at 1-year posttransplant, and then this ratio remained relatively high at least until 4 years posttransplant (Figure S1 in Supplementary Material). In patient #2, the ratio did not change during follow-up, with a profile very similar to that observed in control patients given basiliximab/low-dose RATG induction therapy (Figure 3E). On the other hand, the Treg/memory CD8^+^ T cell ratio increased progressively in patient #3, starting from 1-year posttransplant, reaching very high levels in years 4–6 follow-up (Figure 3F; Figure S1 in Supplementary Material). Instead, in patient #4, the ratio did not undergo relevant variations, remaining at around the mean value of 2, similar to control patients given low-dose RATG induction therapy alone (Figure 3F).

### Immunophenotyping of Peripheral B Cells

**Figure 4:**
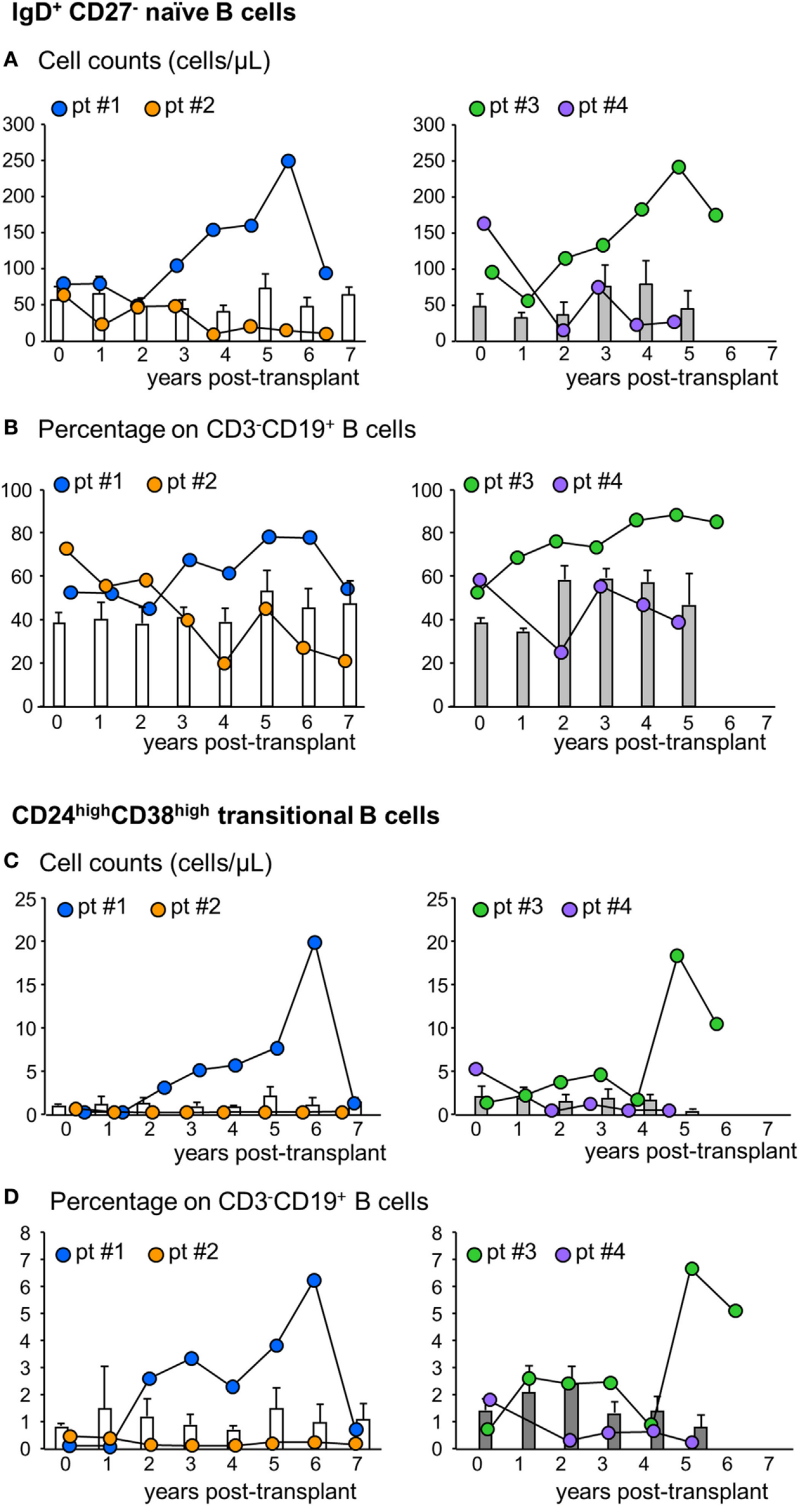
Profile of naïve and transitional B cell counts in the peripheral blood. Number **(A)** and percentages [on total CD3^−^CD19^+^ B cells **(B)**] of naïve IgD^+^CD27^−^ B cells in the peripheral blood during the follow-up in mesenchymal stromal cell (MSC)-treated patients #1 (blue circles) and #2 (orange circles) and in control kidney transplant recipients given basiliximab/low-dose rabbit anti-thymocyte globulin (RATG) induction therapy but not MSC (white histograms) during the follow-up (left panels) and in MSC-treated patients #3 (green circles) and #4 (violet circles) and in control kidney transplant recipients given induction therapy with low-dose RATG alone but not MSC (gray histograms) (right panels). Number **(C)** and percentages [on total CD3^−^CD19^+^ B cells **(D)**] of CD24^high^CD38^high^ transitional B cells in MSC-treated patients #1 and #2 and in control patients given basiliximab/low-dose RATG (white histograms) (left panels) and in MSC-patients #3 and #4 and in control patients given induction therapy with low-dose RATG alone (gray histograms) (right panels), during the follow-up. Data from controls are mean ± SEM, *P* = NS.

Next, we analyzed the phenotype of peripheral B cells according to IgD/CD27 classification (28–30). We found that B cells with a naïve phenotype progressively expanded in the peripheral blood of patients #1 and #3 starting from the third and second year posttransplant, respectively, reaching very high cell counts at year 6 after transplantation (Figure 4A; Figure S1 in Supplementary Material). The expansion of naïve B cells was confirmed by the increase in naïve B cell percentages (Figure 4B). In these two patients, the increase in naïve B cell percentages was paralleled by a decrease in switched B cells (Table S1 in Supplementary Material). By contrast, in patients #2 and #4, naïve B cell counts (Figure 4A) and percentages (Figure 4B) did not show appreciable variations during follow-up, remaining at levels similar to those observed pretransplant and in control patients (Figures 4A,B). As shown in Figures 4C,D, the increase in naïve B cell count in patients #1 and #3 was also paralleled by a marked increase in circulating transitional B cells. Indeed, these B cell counts increased progressively starting from the second year posttransplant in patients #1 and #3, reaching, in the long term, 10-fold higher levels than those observed before transplantation (Figure 4C; Figure S1 in Supplementary Material) and in the other MSC-treated patients and in both control groups, in whom transitional B cells remained at low levels during the entire follow-up period (Figure 4C). The increased transitional B cell counts in patients #1 and #3 were also confirmed by the remarkable increase in the percentages of this B cell subpopulation (Figure 4D). FACS gating strategy and representative dot plots of naïve and transitional B cells are shown in Figure S4 in Supplementary Material.

### *Ex Vivo* Cellular Immune Responsiveness Assays

**Figure 5:**
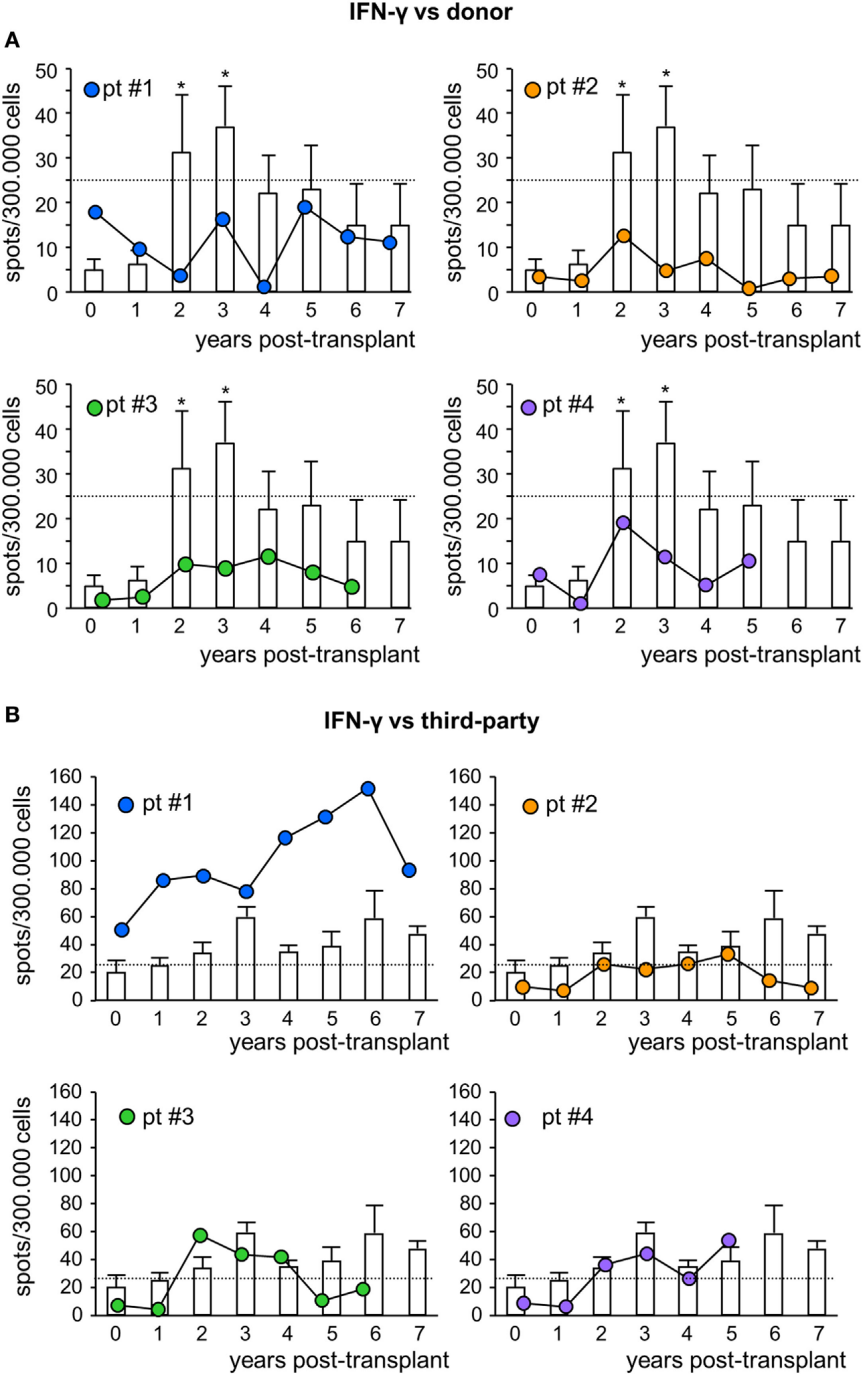
IFNγ ELISpot assay against donor or third-party cells. Frequency of IFNγ-producing T cells after overnight incubation with donor **(A)** or third-party **(B)** cells of peripheral blood mononuclear cells isolated from mesenchymal stromal cell-treated patient #1 (blue circles), patient #2 (orange circles), patient #3 (green circles), and patient #4 (violet circles) and in control kidney transplant patients [white histograms; *n* = 6 given basiliximab/low-dose rabbit anti-thymocyte globulin (RATG) and *n* = 1 given only low-dose RATG induction therapy] during the follow-up. The dotted horizontal line represents the limit of test positivity (25 spots/300.000 responder cells). Data from controls are mean ± SEM; *P < 0.05 vs time 0.

To assess the pro-tolerogenic milieu, MSC-treated and control transplant recipients were monitored for alloimmune response against donor and third-party antigens. In all MSC-treated patients, the frequency of IFNγ-producing memory T cells in response to donor antigens was below the positive threshold of 25 spots/300,000 cells (31, 32) at basal evaluations, and remained below this threshold during the entire follow-up (Figure 5A). However, in control patients, the basal frequency of anti-donor IFNγ-producing memory T cells was below the positive threshold as well, but then tended to increase during long-term follow-up, reaching significantly higher values than pretransplant at years 2 and 3 posttransplant (Figure 5A). The frequency of IFNγ-producing memory T cells in response to third-party cells was more variable (Figure 5B; Figure S5 in Supplementary Material).

**Figure 6:**
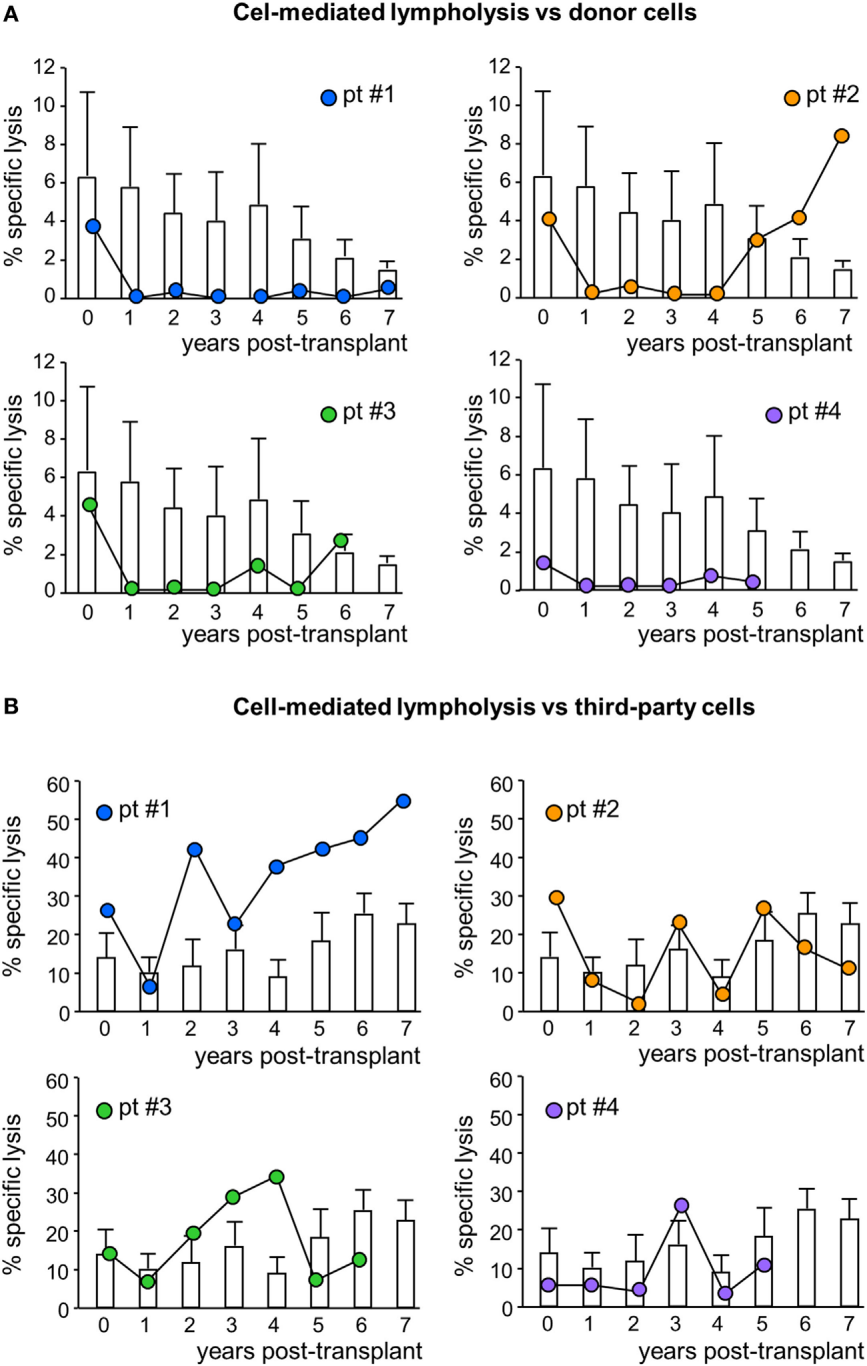
Cell-mediated lympholysis against donor or third-party cells. Percentage of specific lysis of donor **(A)** or third-party **(B)** cells after 4 h incubation of ^51^Cr-labeled donor cells with peripheral blood mononuclear cells isolated from mesenchymal stromal cell-treated patient #1 (blue circles), patient #2 (orange circles), patient #3 (green circles), and patient #4 (violet circles) and in control kidney transplant patients [white histograms; *n* = 6 given basiliximab/low-dose rabbit anti-thymocyte globulin (RATG) and *n* = 1 given only low-dose RATG induction therapy] during the follow-up. Data from controls are mean ± SEM.

In MSC-treated patients, the CD8^+^ T cell-mediated lympholysis of donor cells sharply decreased after transplantation, remaining lower than pretransplant values throughout their respective follow-up, with the exception of patient #2, in whom anti-donor CD8^+^ T cell cytotoxicity resumed basal levels from year 5 posttransplant (Figure 6A; Figure S1 in Supplementary Material). Notably, in control patients, the anti-donor CD8^+^ T cell-mediated lympholysis did not significantly change and remained similar to pretransplant levels during follow-up (Figure 6A). Moreover, in response to third-party cells, the CD8^+^ T cell cytolytic activity increased during the first 3 years posttransplant in patients #1 and #3, and fluctuated in patients #2 and #4 (Figure 6B). In control patients, there were no relevant changes to the CD8^+^ T cell cytolytic response to third-party antigens during the 7 years of follow-up (Figure 6B).

### Immunophenotyping of Peripheral NK Cells, NKT Cells, and Monocytes

The interactions between MSC and NK cells are very complex (33–35). Up to now, however, no experimental studies or clinical evidence are available on the possible effect of MSC on NK cell phenotype and *vice versa* in solid organ transplantation.

**Figure 7:**
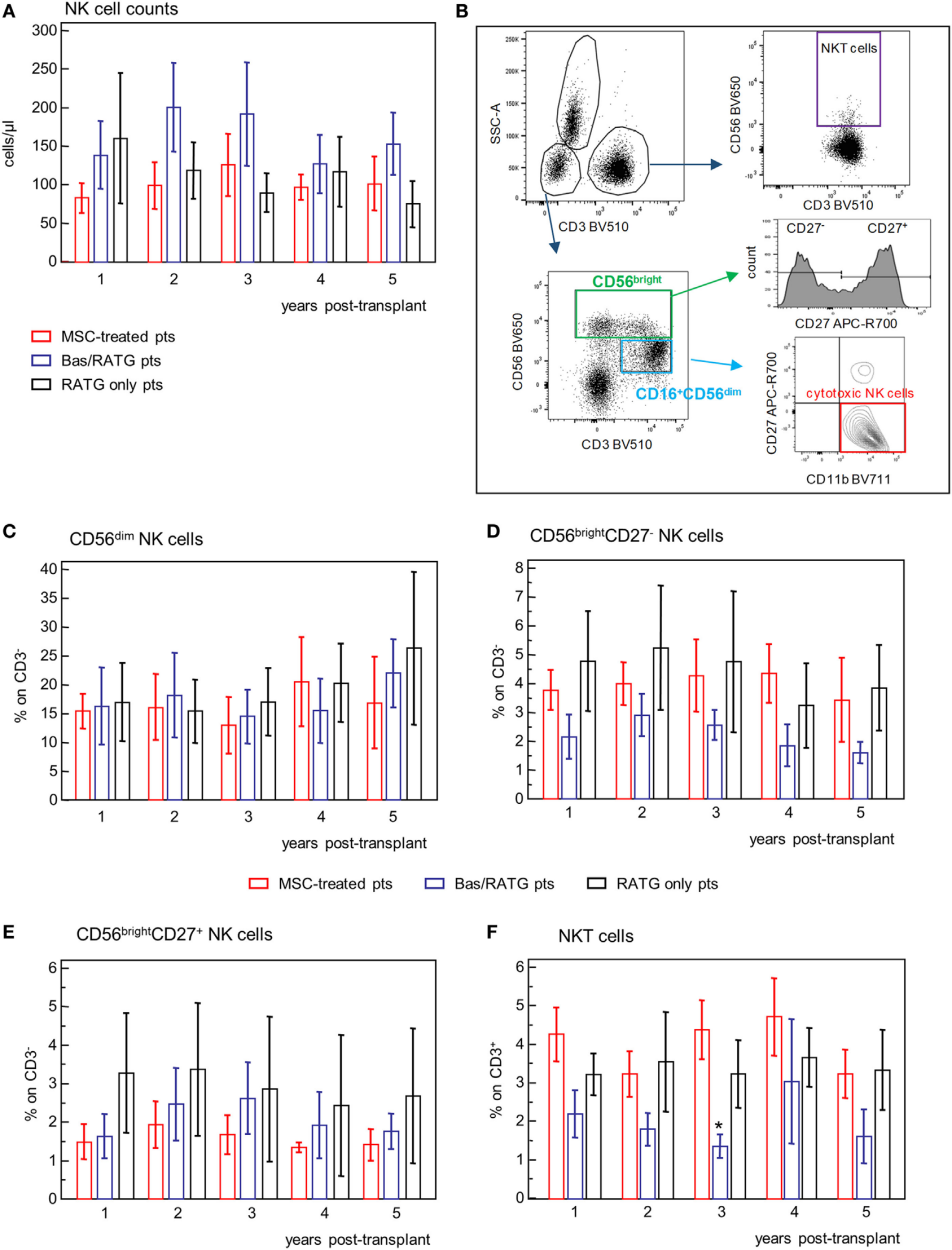
Profile of circulating NK cell subsets. **(A)** Profile of NK cell counts in mesenchymal stromal cell (MSC)-treated patients (*n* = 4), and kidney transplant patients undergoing induction therapy with bas/low dose rabbit anti-thymocyte globulin (RATG) (*n* = 6) or with low-dose RATG alone (*n* = 5) from 1 to 5 years posttransplant. Data are mean ± SEM. *P* = NS. **(B)** Gating strategy for identification of natural killer cell subsets and natural killer T cells. In the CD45^+^ live singlet cells, the population of CD3^−^ lymphocytes was gated on the morphology based on SSC and plotted for CD16 and CD56 expression. CD16^+^CD56^dim^ cells positive for CD11b expression and negative for CD27 were identified as cytotoxic NK cells. The CD56^bright^ CD16^−/+^ NK cells were divided into CD27^+^ and CD27^−^ subpopulations. NKT cells were defined as CD3^+^CD56^+^ cells. The frequency (% on the gated CD3^−^ population) of CD56^dim^ NK cells, CD56^bright^ NK cells negative or positive for CD27 expression, and of NKT cells (% on CD3^+^ cells) in patients given MSC infusion and in kidney transplant patients undergoing induction therapy with bas/low dose RATG or with low-dose RATG alone from 1 to 5 years posttransplant are reported in panels **(C–F)**, respectively. Data are mean ± SEM, *P < 0.05 vs MSC-treated patients and control patients given low-dose RATG at the same time point.

In our MSC-treated patients, circulating NK cell counts fluctuated in the range 100–200 cells/μL, with no significant variation during the 5-year posttransplant follow-up. Similar NK cell counts were shown in control kidney transplant recipient groups (Figure 7A).

Human NK cells are classified into two different subsets based on their expression of CD56 and CD16 with different ability to mediate cytotoxicity and to release cytokines (36). The population of CD56^dim^CD16^+^ NK cells is mostly responsible of cytotoxicity following specific recognition of their targets and is the predominant population in the peripheral blood (36). In our setting, CD56^dim^CD16^+^ were positive for CD11b expression and negative for CD27 and were identified as cytotoxic NK cells (Figure 7B). The CD56^bright^ CD16^−/low^ NK cells, which are the predominant NK cell subset in peripheral lymphoid organs, are the major producer of cytokines (36). Among the CD56^bright^ NK cell subset, the expression of CD27 further divided NK with cytotoxic capacity (CD27^−^) and NK with higher capacity to produce cytokines (CD27^+^) (37) (Figure 7B).

The frequencies of CD56^dim^ NK cell, CD56^bright^ NK cells either positive or negative for CD27 expression were very similar among MSC-treated patients and kidney transplant recipients given basiliximab/low-dose RATG or only low-dose RATG as induction therapy, and did not undergo significant modifications during the follow-up (Figures 7C–E). Similarly, the frequency of NKT cells (Figures 7B,F) during the 5-year follow-up was comparable between MSC-treated patients and control kidney transplant recipients given RATG induction therapy. Percentages of NKT cells in patients given induction therapy with basiliximab/low-dose RATG were lower compared with MSC-treated patients and patients given induction therapy with low-dose RATG alone at each time point analyzed, reaching statistical significance 3 years after transplantation.

Overall, our analysis showed that MSC cell therapy did not have a major impact on NK cell counts and phenotype and on frequency of NKT cells on the long-term posttransplant.

**Figure 8:**
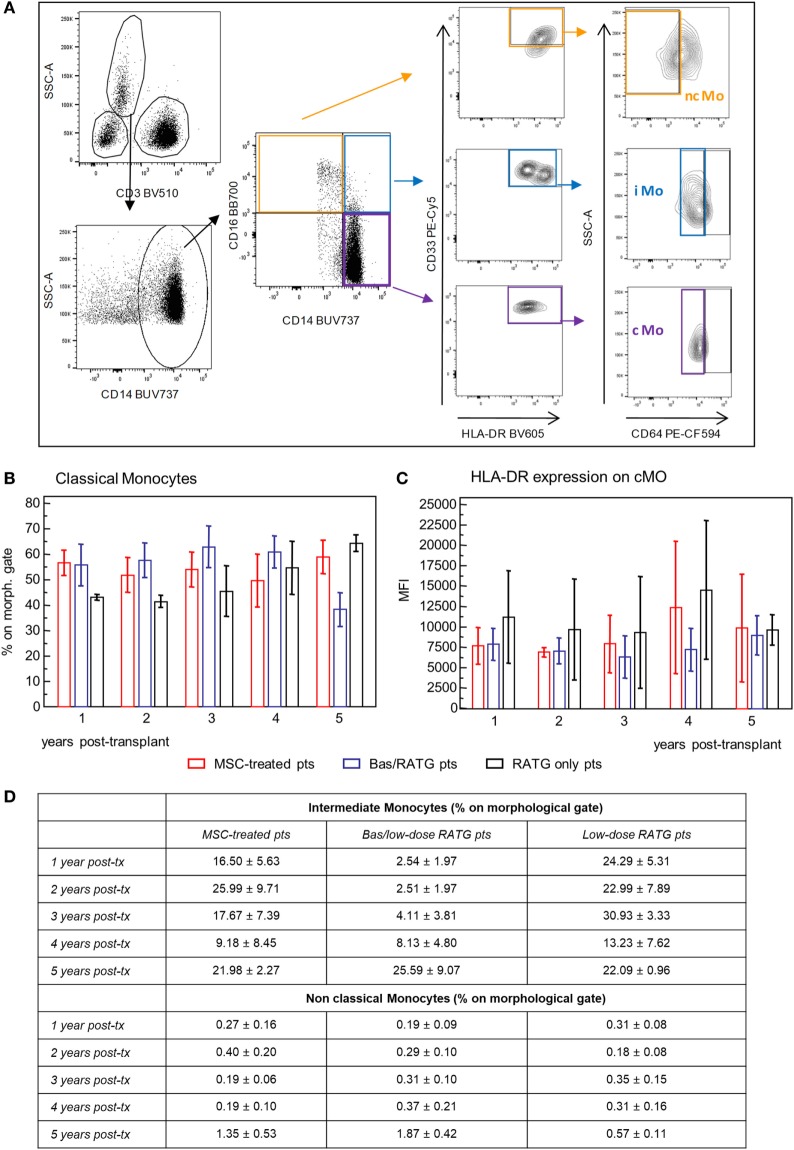
Profile of circulating monocytes. **(A)** Gating strategy for identification of classical, non-classical, and intermediate monocytes in peripheral blood mononuclear cells. In the CD45^+^ live singlet cells, the population of CD3^−^ leukocytes was gated on the morphology based on SSC and plotted for CD14 expression. Classical monocytes were identified as CD64^+^ cells on gated CD33^+^HLADR^+^ cells in the CD14^+^CD16^−^ cell population, intermediate monocytes as CD64^+^ cells on gated CD33^+^HLADR^+^ in the CD14^+^CD16^+^ cell population, and non-classical monocytes as CD64^−^ cells on gated CD33^+^HLADR^+^ cells in the CD14^−^CD16^+^ cell population. Percentages of classical monocytes (% on the gated CD3^−^ population) and their HLA-DR expression in patients given mesenchymal stromal cell (MSC) infusion (*n* = 4) and in kidney transplant patients undergoing induction therapy with bas/low dose rabbit anti-thymocyte globulin (RATG) (*n* = 6) or with low-dose RATG alone (*n* = 5) from 1 to 5 years posttransplant are reported in panels **(B,C)**, respectively. Data are mean ± SEM, *P* = NS. Table in panel **(D)** reported mean ± SEM of intermediate and non classical monocytes; *P* = NS.

We also examined the phenotypic profile of circulating monocytes, which are classified on the basis of relative expression of CD14 and CD16 surface markers in classical monocytes (CD14^++^CD16^−^), intermediate monocytes (CD14^++^CD16^+^), and non-classical monocytes (CD14^+^CD16^++^) (38) with different functions. Classical monocytes are phagocytic cells and preferentially express genes involved in angiogenesis, wound healing, and coagulation, intermediate monocytes are pro-inflammatory and with the capability of antigen presentation and T cell activation, while non-classical monocytes display patrolling properties. These subsets expressed high levels of HLA-DR and CD33 and different levels of CD64 (39, 40) (Figure 8A). Very few studies are available on the possible interaction between MSC and monocytes (41, 42). The frequency of classical monocytes in our MSC-treated patients was around 70% during the follow-up and similar to that found in kidney transplant recipients in the two control groups (Figure 8B). The expression of HLA-DR on this monocyte subset ranged around value of MFI of 7,500 in MSC-treated and control transplant patients. HLA-DR expression on classical monocytes remained constant during the follow-up in the three groups of patients (Figure 8C). The frequencies of intermediate and non-classical monocyte were more variable among and between subjects than classical monocytes, with no significant differences during the 5-year follow-up in the three groups of kidney transplant recipients (Figure 8D).

Therefore, MSC infusion in kidney transplant patients did not associate with significant changes of the phenotype of circulating monocytes in the long-term.

### *De Novo* DSA Development After Kidney Transplantation

All MSC recipients and control patients given basiliximab/low-dose RATG induction therapy were negative for DSA at the time of kidney transplantation. Patient #1 developed *de novo* class I DSA against B57 (MFI: 5,740) and B58 (MFI: 5,590) antigens 7 years after transplantation (Figure S1 in Supplementary Material). Notably, DSA were never detected in the remaining MSC recipients throughout their respective follow-up. By contrast, three of six control patients were transiently positive for *de novo* class II DSA reactive to DQ and/or DR antigens during the 7-year observation period. In particular, in a patient DSA were detected at years 4 (anti-DQ6, MFI: 6,800) and 5 (anti-DQ6, MFI: 5,600; anti-DR0404, MFI: 2,730; anti-DR16, MFI: 2,760) after transplantation. A second patient tested positive for DSA at years 2 (anti-DQ6, MFI: 3,000) and 5 (anti-DQ5, MFI: 2,690), while in a third patient DSA were identified in a single occasion 5 years after transplantation (anti-DQ0603, MFI: 3,260).

### Phased Withdrawal of CsA Therapy

Patient #3 had a favorable posttransplant clinical course with evidence of sustained pro-tolerogenic immune milieu. In particular, no features of subclinical rejection were observed in the 1-year protocol biopsy (11), and graft function remained stable for 2 years after transplantation (measured GFR: 67.07 mL/min/1.73 m^2^), without evidence of *de novo* DSA development. At this point, the patient exhibited a pro-tolerogenic profile characterized by an increased ratio of Treg/memory CD8^+^ T cell percentage and expansion of naïve and transitional B cells in the peripheral blood, along with suppression of donor-specific CD8^+^ T cell cytotoxicity. In light of these clinical and immunological findings, we chose to attempt to wean patient off immunosuppressive therapy while monitoring renal graft function closely. Accordingly, the CsA dose was gradually tapered starting from month 26 and was eventually discontinued at month 73 posttransplant (Figure S1 in Supplementary Material), leaving the patient on low-dose MMF monotherapy since then. No clinical or laboratory evidence of rejection was observed during or after CsA withdrawal. Graft function remained normal and actually improved over time, as demonstrated by measured GFR, which increased by 2.30 mL/min/1.73 m^2^/year over 6 years of follow-up. *De novo* DSA were never detected in serum samples, even after CsA was discontinued. For safety reasons, immunosuppressive therapy weaning was not attempted in the other MSC recipients due to either transient acute renal insufficiency or acute cellular rejection early after transplantation, even though signs of immunoregulation were observed in patient #1.

### Pretransplant Infusion of MSC in the Setting of Induction Therapy With Basiliximab/Low-Dose RATG

**Figure 9:**
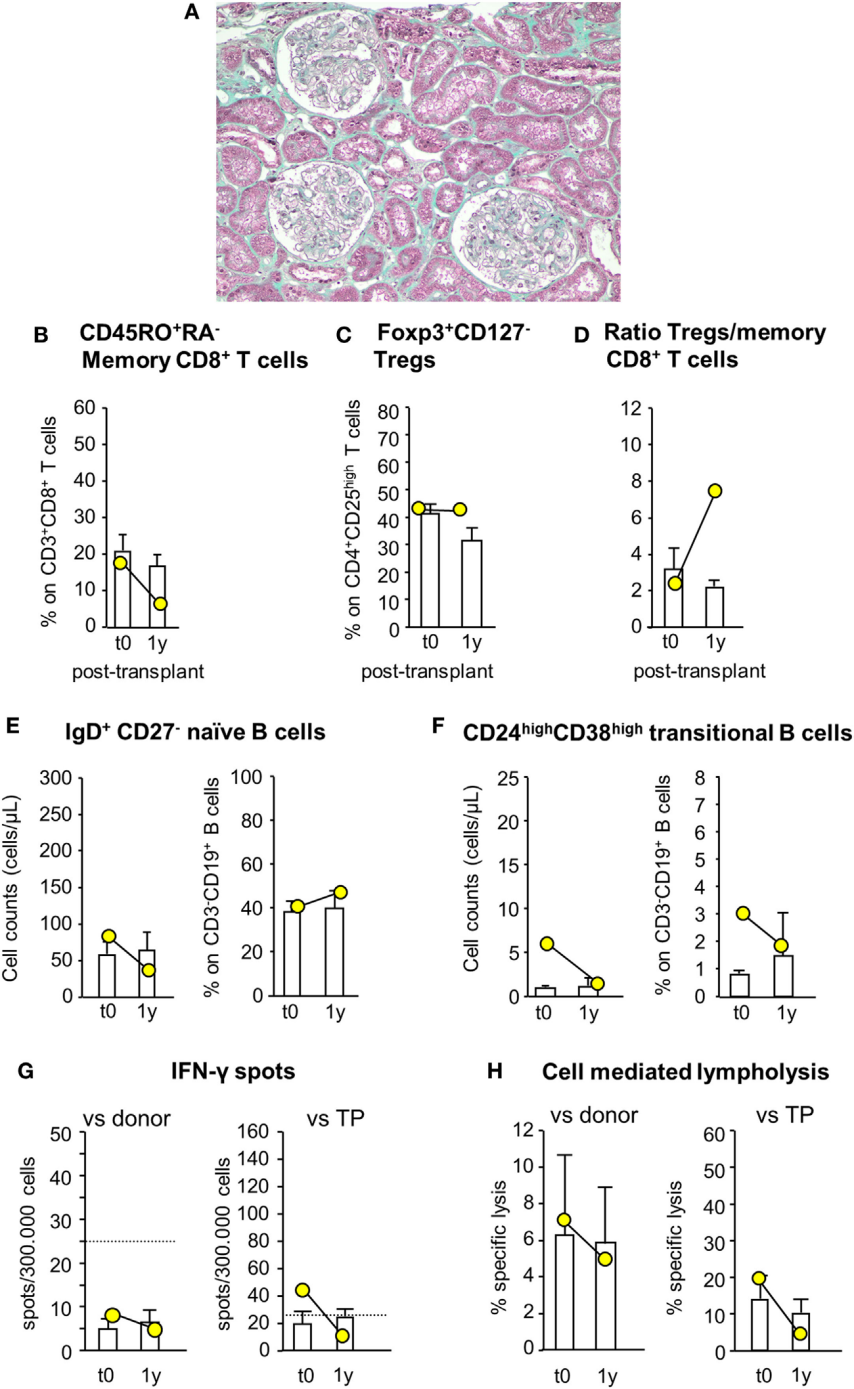
Histologic analysis of protocol kidney graft biopsy, profile of circulating B and T cell subsets, and immunologic functional assays of memory T cell response in patient #5, who received pretransplant infusion of mesenchymal stromal cell (MSC) in the setting of induction therapy with basiliximab/low-dose rabbit anti-thymocyte globulin (RATG). Patient #5, a 42-year-old woman with end-stage renal disease secondary to tubular interstitial nephropathy, received a kidney transplant from her sister, mismatched for two HLA alleles. She was given MSC before surgery (day −1) and induction therapy with the combination of basiliximab and low-dose RATG. A representative image of Gomori’s trichrome staining on 1-year protocol kidney graft biopsy (original magnification 100×) of MSC-treated patient #5 is shown **(A)**. Percentages of circulating CD45RO^+^RA^−^ memory CD8^+^ T cells (on CD3^+^CD8^+^ T cells) **(B)**, percentages of circulating regulatory T cells (Tregs) (Foxp3^+^CD127^−^ on CD4^+^CD25^high^ T cells) **(C)**, and ratio of the percentages of Tregs to CD45RO^+^RA^−^ memory CD8^+^ T cells **(D)** in patient #5 (yellow circles) and in control kidney transplant recipients given basiliximab/low-dose RATG induction therapy but not MSC (white histograms) from baseline (pretransplant) to 1-year posttransplant. Number of circulating naïve IgD^+^CD27^−^ B cells **(E)** and CD24^high^CD38^high^ transitional B cells **(F)** in patient #5 (yellow circles) and in control kidney transplant recipients given basiliximab/low-dose RATG induction therapy (white histograms) from baseline to 1-year posttransplant. ELISpot for IFNγ **(G)** and CD8^+^ T cell function by T cell-mediated lympholysis [as percentage of specific lysis at 50:1 effector-target ratio **(H)**] toward donor or third-party antigens in patient #5 (yellow circles) and in control kidney transplant recipients given basiliximab/low-dose RATG induction therapy (white histograms) on peripheral blood mononuclear cells collected pretransplant and at 1 year after transplantation. Data are mean ± SEM.

Since patient #4 developed acute cellular rejection 2 weeks after kidney transplantation, to limit the risk of early posttransplant acute rejection the study protocol was modified again for patient #5, who still received MSC before surgery (day −1) but was given both basiliximab and RATG as induction therapy. Patient #5 was a 42-year-old woman on hemodialysis due to ESRD secondary to tubular interstitial nephropathy. She received a renal transplant from her sister, mismatched for two HLA alleles (one mismatch on HLA-B and one on HLA-DR, while HLA-A alleles were coincidental). In this patient, pretransplant infusion of MSC was uneventful. Renal function gradually recovered and normalized within the first two post-operative weeks (serum creatinine: 1.32 mg/dL). Thereafter, graft function remained relatively stable up to 12 months posttransplantation (serum creatinine: 1.52 mg/dL, measured GFR: 52.59 mL/min/1.73 m^2^; proteinuria: 0.09 g/24 h). At this time, a protocol biopsy showed mild mesangial expansion and patent capillary lumina surrounded by well-preserved tubulointerstitial compartment (Figure 9A).

Immunophenotypic analysis of peripheral blood lymphocytes showed that the proportion of memory CD8^+^ T cells was reduced by almost 70% 1 year after transplantation compared with pretransplant value, whereas Treg percentage did not change over the same period (Figures 9B,C). Thus, the ratio of Treg/memory CD8^+^ T cells markedly increased 1 year following transplant compared with basal level (Figure 9D). Circulating naïve and transitional B cell counts and percentages did not change appreciably 1-year posttransplant compared with pretransplant values (Figures 9E,F). Regarding functional immunological studies, the basal frequency of IFNγ-producing memory T cells in response to donor antigens was lower than the positive threshold of 25 spots/300,000 cells, and tended to reduce 1 year after transplantation (Figure 9G). Instead, the frequency of IFNγ-producing memory T cells in response to third-party cells exceeded the positive threshold at baseline evaluation, but it fell below the above indicated threshold 1-year posttransplant (Figure 9G). At this time, CD8^+^ T cell-mediated lympholysis against donor and third-party antigens decreased compared with pretransplant values (Figure 9H).

## Discussion

We report here that inducing long-term (up to 5–7 years) stable renal graft function under minimization of maintenance immunosuppressive therapy can be achieved after peri-transplant single infusion of autologous bone marrow-derived MSC. Moreover, we documented that MSC infusion was neither associated with increased susceptibility to infections, nor tumor development in the long-term. These findings extend the established evidence of an early good safety profile for MSC therapy (10) to very long-term posttransplant follow-up, and strengthen it, even with prolonged pharmacologic immunosuppression.

The results of this study also provided the first insights into the long-term *in vivo* effects of MSC infusion on peripheral lymphocyte subsets with effector and regulatory properties, as well as on host T cell allo-reactivity. In particular, we showed that in most MSC recipients, but not in the control transplant patients, the percentage of circulating memory CD8^+^ T cells decreased markedly after surgery, remaining lower than pretransplant values for at least 5 years follow-up. Interestingly, the change in memory CD8^+^ T cell profile was coupled with a profound and persistent reduction in *ex vivo* donor-specific CD8^+^ T cell cytotoxicity. These findings indicate that MSC can control memory CD8^+^ T cell proliferation and donor-specific CD8^+^ T cytolytic function long-term, effects not shared by current immunosuppressive drugs (43). It is widely held that, in alloimmunity, the strength of the allogeneic response is dictated by the relative balance between regulatory and effector/memory T cells (44). In patients #1 and #3, but not in the other MSC recipients or in kidney transplant patients of either control group, the ratio of Treg/memory CD8^+^ T cell percentage increased after transplantation, remaining higher than pretransplant values for at least 4 years of follow-up, indicating a skew in the host immune response toward regulation. Notably, in these very patients the pool of circulating naïve and transitional B cells expanded over the long-term posttransplantation strikingly, indicating the emergence of a B cell profile consistent with that previously identified as part of the tolerogenic signature in immunosuppression-free kidney transplant recipients (45–49). MSC infusion did not associate with relevant long-term modification in the frequency of peripheral NK cell and monocyte subsets. Together, these findings suggest that in some patients MSC trigger an active peripheral immune regulatory process following infusion that later self-sustains. Actually, a B cell phenotype in MSC recipients similar to that associated with operational kidney transplant tolerance provides a potential marker of MSC-induced immunomodulation that may anticipate a pro-tolerogenic milieu.

*De novo* posttransplant development of DSA is now recognized as one of the leading causes of long-term allograft failure (50–52). Intriguingly, *de novo* DSA were detected less frequently and much later in MSC-treated patients than in control kidney transplant recipients given the same induction therapy without cell infusion. Consistently, in animal models of renal transplantation MSC infusion was associated with reduced levels of circulating donor-specific antibodies (53, 54). These clinical and experimental findings reflect *in vitro* evidence showing that MSC can suppress allo-specific antibody production (55), indicating that MSC have a protective effect on DSA development. Actually, the absence of *de novo* DSA during the early posttransplant period in MSC recipients may account for the trend toward lower rate of GFR decline in the long-term after transplantation compared with that reported in control kidney transplant patients not given cell infusion.

Although this clinical study was not designed primarily to assess the efficacy of MSC therapy to induce tolerance in living-donor kidney transplant recipients, the clinical and immunological features of patient #3 guided the decision to gradually wean patient off immunosuppressive therapy. The patient successfully discontinued CsA without evidence of rejection or *de novo* DSA development, and is currently in the middle of MMF dose tapering in anticipation of complete immunosuppressive drug withdrawal. It is conceivable that MSC therapy did result in a milieu permissive to graft tolerance developing in this patient, so that CsA could safely be phased out.

These findings, though limited to a single MSC-treated patient, are remarkable because so far most attempts to withdraw calcineurin inhibitors from kidney transplant recipients have been associated with high rates of acute rejection and/or *de novo* DSA development (56–62). Even when performed in carefully selected cohorts of stable kidney transplant recipients deemed to be at low immunological risk based on clinical and histological criteria, withdrawal of calcineurin inhibitors was successful only in 6 of 14 patients, in spite of continuation of maintenance immunosuppressive therapy with steroids and MMF (63). Although six patients of this cohort successfully discontinued calcineurin inhibitors, the development of a pro-tolerogenic state with this minimization protocol could not be anticipated, since they were still on dual immunosuppressive regimen with steroids and MMF. At variance, in our patient we reasonably expect a pro-tolerogenic state is in place since signs of immunoregulation have been consistently and sustainably detected in *ex vivo* immunomonitoring and he is currently on the tapering phase of the low-dose MMF monotherapy with stable renal function.

Unfortunately, not all kidney transplant recipients who were given MSC therapy consistently had signs of immunoregulation. The reason(s) for these patients not harboring a common immunological profile following peri-transplant infusion of the same dose of autologous MSC, isolated and expanded under identical cultural conditions, remains ill-defined. This can be understood by considering that MSC preparations display substantial donor-to-donor variability in their capability to dampen the alloimmune response *in vitro*, despite common immunophenotype and tri-lineage differentiation potential (64). A complementary explanation could be the nature of the host microenvironment surrounding MSC, which may have influenced their polarization toward a tolerogenic or inflammatory phenotype (65). Intriguingly, apoptosis of MSC and engulfment of phagocytes with apoptotic MSC have been recently found to be essential steps to initiate MSC-induced immunosuppression (66). In patients with graft-vs-host disease, high cytotoxic activity of recipient cytotoxic cells to induce *ex vivo* MSC apoptosis was associated with clinical response to MSC (66), indicating that this evaluation could be a potential biomarker to predict clinical response to MSC and to stratify patients for this treatment. Together, the above observations refute the reliability of testing MSC-based therapy as a strategy for promoting graft tolerance in unselected cohorts of organ transplant recipients within the framework of scheduled immunosuppression withdrawal protocols, just based on clinical criteria. Indeed, a recent study in liver transplant recipients who were given posttransplant MSC infusion showed that immunosuppression withdrawal attempt based on clinical criteria, but in the absence of Treg expansion, was not successful (67). Thus, we would like to propose applying a comprehensive immune monitoring approach, including T- and B-cell subset phenotyping and *ex vivo* functional assays, to demonstrate the efficacy of MSC in promoting donor-specific immunoregulation and developing a pro-tolerogenic environment in organ transplant recipients, which would provide robust criteria for identifying the most suitable candidates for minimization or even withdrawal of immunosuppressive therapy at a given time after transplantation.

We acknowledge the limitations of the sample size of the present study. Indeed, as a phase 1 study with a limited number of participants, the study was not powered to detect improvements in kidney graft outcomes after autologous MSC infusion. Nevertheless, studying a small cohort of patients very extensively using cutting-edge immunological methods enabled us to draw a mechanistic picture of how MSC can influence the host immune response long-term after kidney transplantation. Moreover, these findings in transplant recipients of kidneys from a living-related donor may not necessarily translate to a deceased kidney transplant program. However, the know-how we have gained in recent years regarding the safety and biologic/mechanistic effects of bone marrow-derived MSC in living-donor kidney transplant recipients, allowed us to devise and conduct a clinical trial in deceased-donor kidney transplant patients using the same design structure (ClinicalTrials.gov: NCT02565459).

In conclusion, our clinical study has shown that peri-transplant infusion of autologous bone marrow-derived MSC to living-donor HLA-mismatched kidney transplant recipients under low-dose maintenance immunosuppressive drugs is safe and without major side effects even over long-term follow-up. MSC treatment promoted, at least in some patients, the development of a long-term pro-tolerogenic environment that could contribute to long-term stabilization of renal allograft function and could possibly determine the selection of MSC-treated transplant recipients for safe withdrawal of maintenance immunosuppressive drugs. Non-invasive immune monitoring tools should be further applied to assess MSC ability to foster donor-specific immunomodulation in solid organ transplantation, to identify patients amenable to safe immunosuppressive drug discontinuation before widespread application of this cell-based technology for tolerance induction.

## Ethics Statement

All treatment protocols were approved by the Istituto Superiore di Sanità [ISS, Rome, authorization number no. 45253(06)-PRE.21-882 and no. 28689(13)321-1223] and by Agenzia Italiana del Farmaco (AIFA) on October 10, 2007 and September 30, 2013, respectively, and by the Institutional Review Board of the Ospedali Riuniti/Azienda Ospedaliera Papa Giovanni XXIII of Bergamo (authorization no. 352, March 18, 2008 and no. 110/13, November 6, 2013). The study is registered with ClinicalTrials.gov (NCT00752479 and NCT02012153). Written informed consent was obtained from all recipients and living donors in accordance with the Declaration of Helsinki.

## Author Contributions

Conception and design of the study: NP, FC, MI, and GR. Acquisition, analysis, and interpretation of data: NP, FC, MT, MC, EG, VP, MM, FG, AV, SF, and EL. Manuscript preparation: MC, NP, FC, and GR.

## Conflict of Interest Statement

The authors declare that the research was conducted in the absence of any commercial or financial relationship that could be construed as a potential conflict of interest.

## Abbreviations

CsA, ciclosporin; DSA, donor-specific anti-HLA antibodies; ESRD, end-stage renal disease; GFR, glomerular filtration rate; HLA, human leukocyte antigen; MSC, mesenchymal stromal cells; MFI, mean fluorescence intensity; MMF, mycophenolate mofetil; PBMCs, peripheral blood mononuclear cells; RATG, rabbit anti-thymocyte globulin; Tregs, regulatory T cells.
