# Supplementary material for: Long-Term Clinical and Immunological Profile of Kidney Transplant Patients Given Mesenchymal Stromal Cell Immunotherapy
Source: Front Immunol. 2018 Jun 14;9:1359. doi: 10.3389/fimmu.2018.01359 (PMC6014158; doi:10.3389/fimmu.2018.01359)
Supplement: Supplementary file 1 [file table_1.PDF]

Supplementary table 1. Percentages and counts of switched, unswitched and double negative B cells (according to IgD-CD27 expression[a]) in control groups and in MSC-treated patients during the follow-up.

	PRE-TX	1 y	2y	3y	4y	5y	6y	7y
**Percentage on $CD19^+$ B cells**								
**Switched B cells (IgD^-CD27^+)**								
Bas/RATG ctr pts	21,7 ± 5,4	23,8 ± 5,1	30,5 ± 4,9	27,0 ± 5,6	34,1 ± 7,5	24,0 ± 6,1	27,6 ± 8,3	26,8 ± 8,3
RATG control pts	19,9 ± 3,4	21,8 ± 3,1	20,4 ± 3,5	23,6 ± 2,9	24,9 ± 3,9	28,8 ± 8,5		
MSC #1	14	14,5	37,5	15	22,2	27,7	10,4	10,4
MSC #2	4,5	32,6	15,4	32,2	44,6	35,4	45,6	45,8
MSC #3	24,2	4,7	8	10,1	4,4	3,2	4,8	
MSC #4	11,6	13,6	43,3	27,7	29,1	30,8		
MSC #5	36,4	23,3						
**Unswitched B cells (IgD^+CD27^+)**								
Bas/RATG ctr pts	17,9 ± 4,4	20,2 ± 3,6	10,4 ± 2,2	8,1 ± 0,8	7,6 ± 0,9	5,7 ± 0,9	9,8 ± 0,6	8,9 ± 2,1
RATG control pts	30,6 ± 3,4	36,4 ± 2,7	7,5 ±1,4°	6,8 ± 1,5°	6,9 ± 1,5°	10,9 ± 1,9		
MSC #1	14,05	23,6	4,5	4,1	3,7	3,0	5,9	10,8
MSC #2	15,5	5,3	8,8	7,6	4,3	7,6	15,0	12,1
MSC #3	8,9	16	2,9	4,7	3,3	5,4	5,8	
MSC #4	21	13,6	6,6	9,3	14,4	21,3		
MSC #5	7,1	4,9						
**Double negative (IgD^-CD27^-)**								
Bas/RATG ctr pts	19,8 ± 3,9	14,8 ± 3,4	16,7 ± 2,3	17,5 ± 3,5	17,4 ± 3,4	17,4 ± 3,3	14,2 ± 3,7	18,2 ± 3,1
RATG control pts	11,6 ± 2,3	7,6 ± 2,0	13,8 ± 4,1	20,6 ± 9,4	20,7 ± 8,2	13,9 ± 4,6		
MSC #1	11	8,9	13,1	12,7	12	9,6	4,8	11,6
MSC #2	7,6	6,8	17,1	19,4	32	11,8	12,3	20,8
MSC #3	14,7	10,2	12,7	11,4	5,3	2,4	3,6	
MSC #4	6,4	19,8	25,3	7,9	10,2	9,5		
MSC #5	15	24,9						
**Absolute counts /cells/μl)**								
**Switched B cells (IgD^-CD27^+)**								
Bas/RATG ctr pts	25,1 ± 4,3	29,7 ± 5,7	40,4 ± 13,3	25,0 ± 6,6	34,6 ± 10,5	27,9 ± 6,0	34,5 ± 13,8	41,6 ± 16,3
RATG control pts	19,9 ± 4,6	20,3 ± 5,8	10,8 ± 2,8	31,5 ± 10,8	33,5 ± 11,9	51,8 ± 37,0		
MSC #1	19,74	22,19	41,25	23,25	55,72	55,95	33,28	18,20
MSC #2	3,92	12,71	12,47	37,67	21,41	13,81	18,24	13,28
MSC #3	45,01	3,71	12,24	18,58	9,20	8,80	9,65	
MSC #4	33,18	21,22	22,52	37,40	12,22	18,17		
MSC #5	71,71	17,94						
**Unswitched B cells (IgD^+CD27^+)**								
Bas/RATG ctr pts	23,1 ± 5,9	27,7 ± 6,2	16,5 ± 8,0	8,5 ± 2,2	8,0 ± 1,9	7,2 ± 1,5	10,6 ± 1,8	12,5 ± 2,7
RATG control pts	39,6 ± 15,6	34,7 ± 8,3	4,7 ± 1,9°	9,0 ± 4,1	8,7 ± 3,8	17,3 ± 11,7		
MSC #1	19,8105	36,108	4,95	6,355	9,287	6,06	18,88	18,9
MSC #2	13,485	2,067	7,128	8,892	2,064	2,964	6	3,509
MSC #3	16,554	12,64	4,437	8,648	6,897	14,85	11,658	
MSC #4	60,06	21,216	3,432	12,555	6,048	12,567		
MSC #5	13,987	3,773						
**Double negative (IgD^-CD27^-)**								
Bas/RATG ctr pts	27,8 ± 10,3	27,8 ± 15,8	22,4 ± 6,5	20,7 ± 9,1	17,7 ± 3,9	20,8 ± 3,2	18,1 ± 6,7	27,5 ± 7,6
RATG control pts	11,1 ± 2,2	6,9 ± 2,5	7,1 ± 2,2	33,3 ± 22,4	28,1 ± 15,4	25,4 ± 18,9		
MSC #1	15,51	13,62	14,41	19,69	30,12	19,39	15,36	20,30
MSC #2	6,61	2,65	13,85	22,70	15,36	4,60	4,92	6,03
MSC #3	27,34	8,06	19,43	20,98	11,08	6,60	7,24	
MSC #4	18,30	30,89	13,16	10,67	4,28	5,61		
MSC #5	29,55	19,17						
° P<0.05 vs pre-tx								

[a] Klein U, Rajewsky K and Ku¨ppers R: Human immunoglobulin (Ig)M+IgD+ peripheral blood B cells expressing the CD27 cell surface antigen carry somatically mutated variable region genes: CD27 as a general marker for somatically mutated (memory) B cells. *J Exp Med* 188: 1679–1689, 1998
