# Supplementary figures and images for: Long-Term Clinical and Immunological Profile of Kidney Transplant Patients Given Mesenchymal Stromal Cell Immunotherapy
Source: Front Immunol. 2018 Jun 14;9:1359. doi: 10.3389/fimmu.2018.01359 (PMC6014158; doi:10.3389/fimmu.2018.01359)

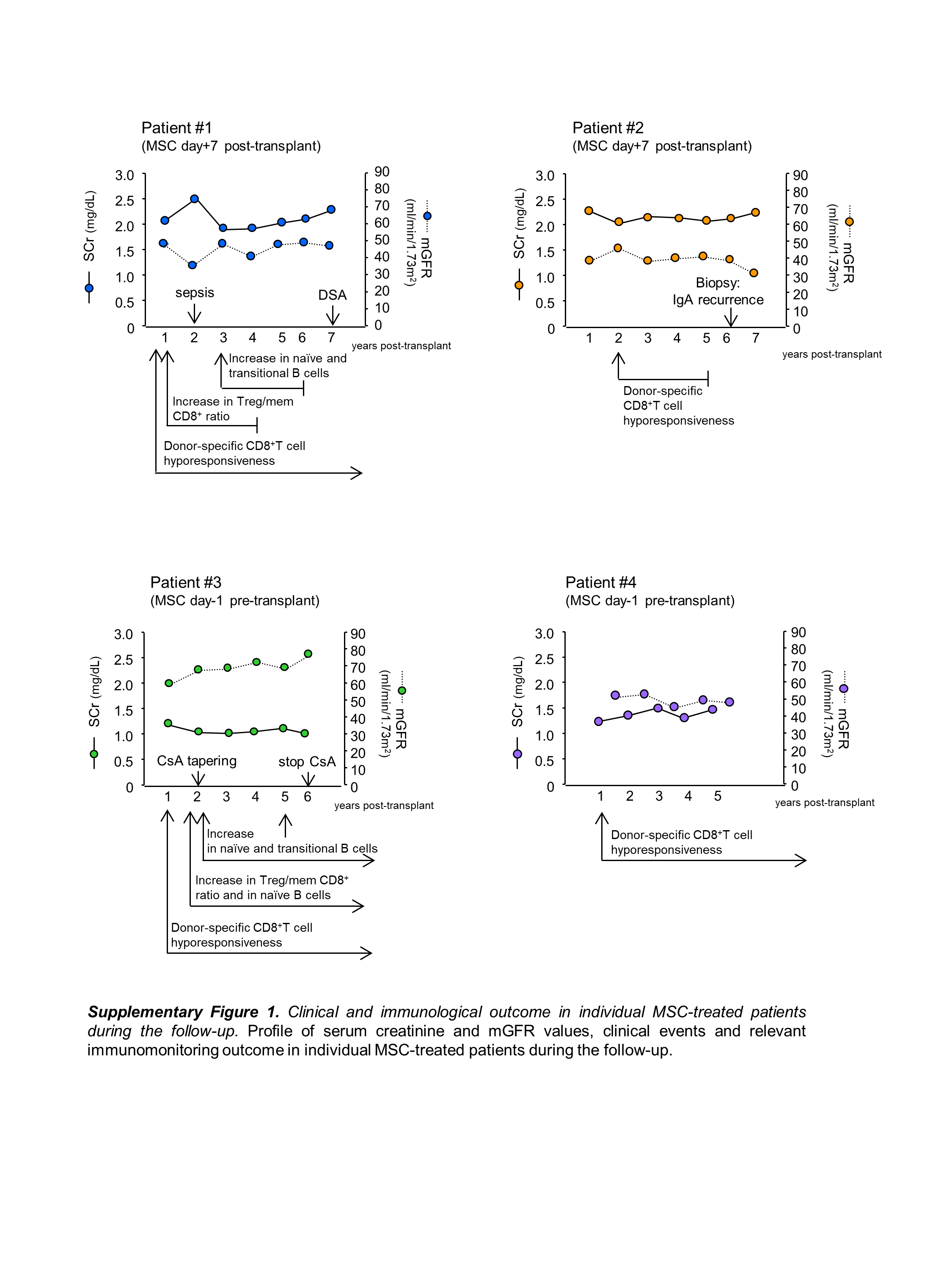

Supplement: Supplementary file 2 [file image_1.tif]

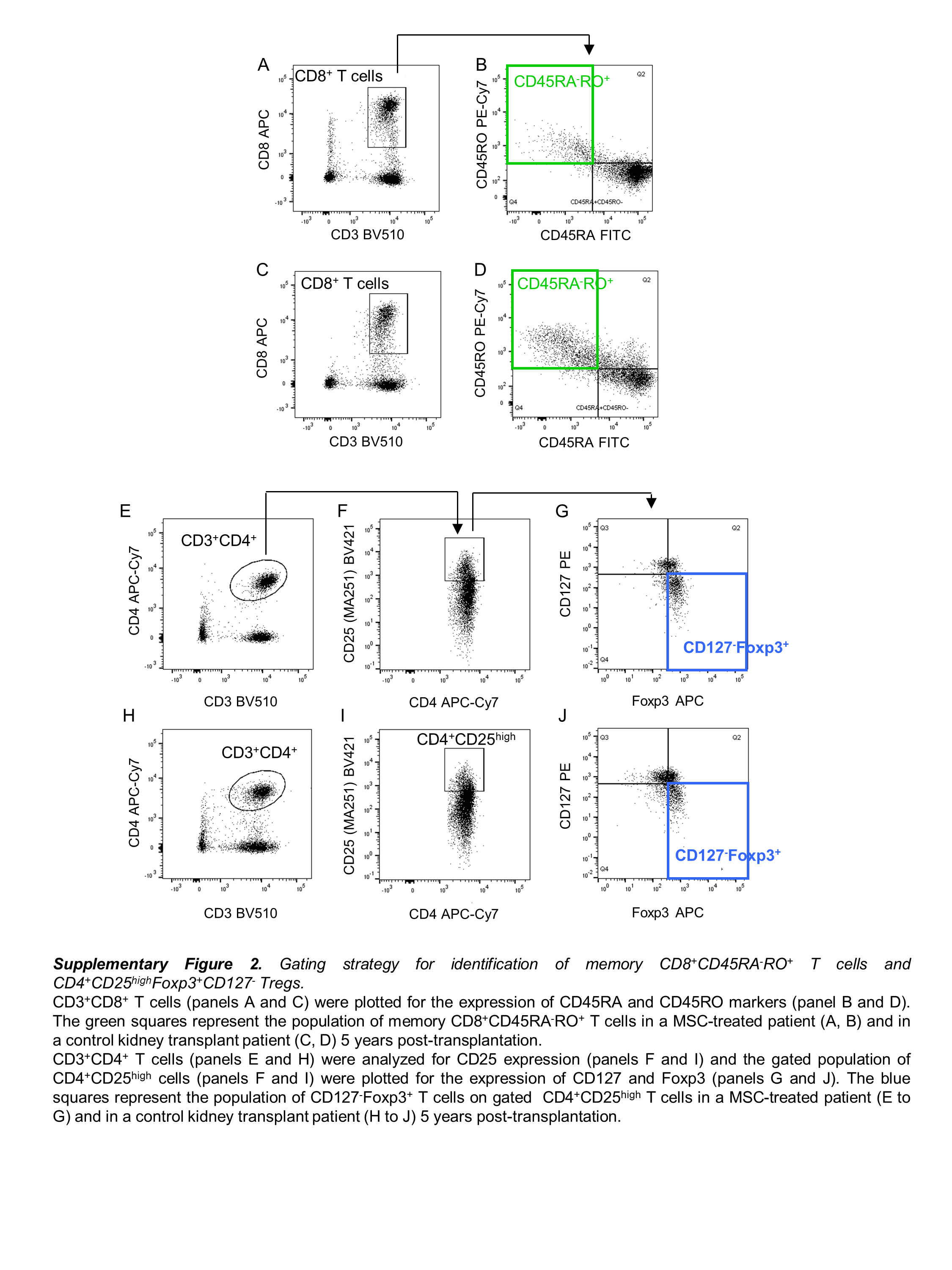

Supplement: Supplementary file 3 [file image_2.tif]

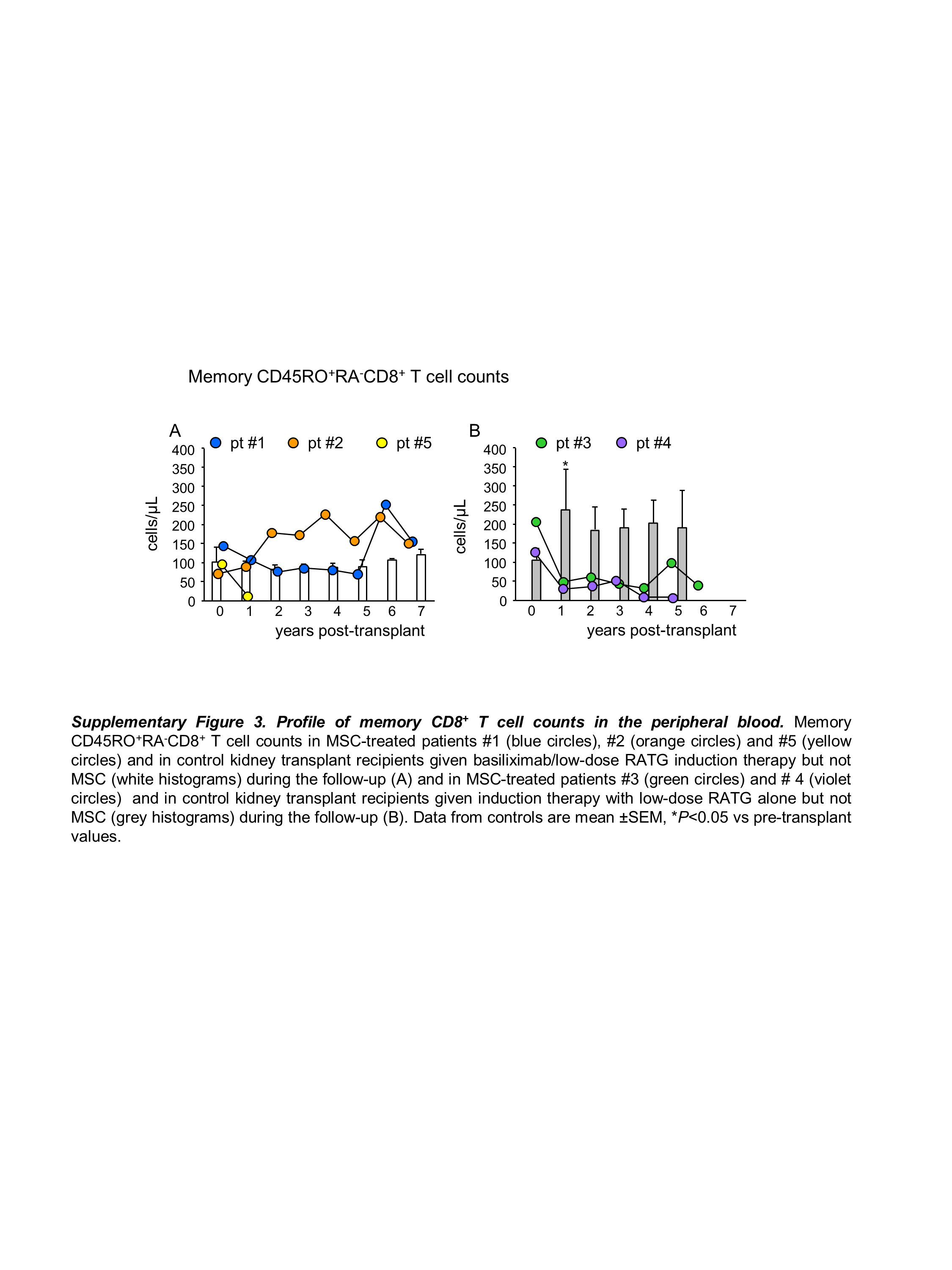

Supplement: Supplementary file 4 [file image_3.tif]

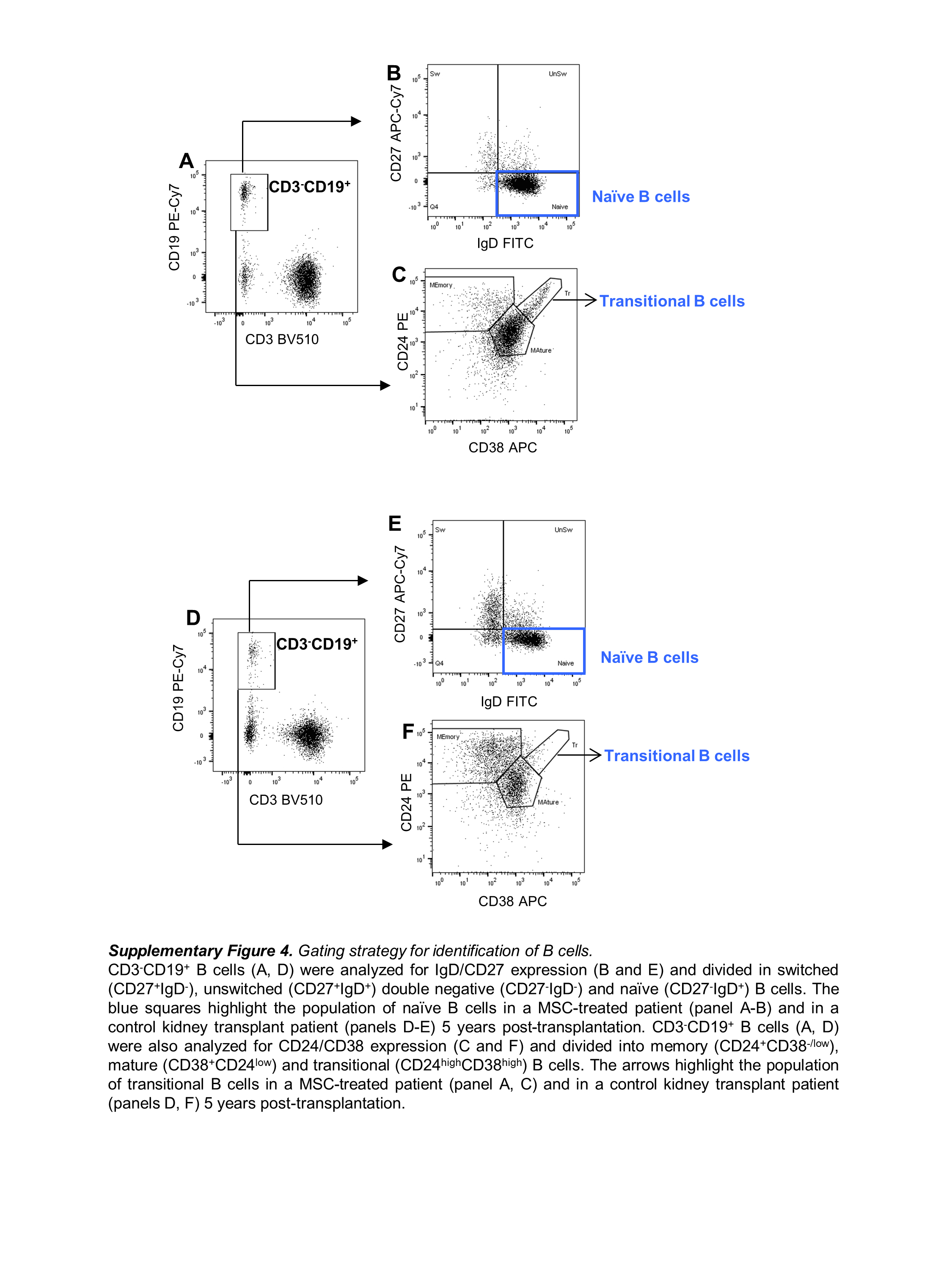

Supplement: Supplementary file 5 [file image_4.tif]

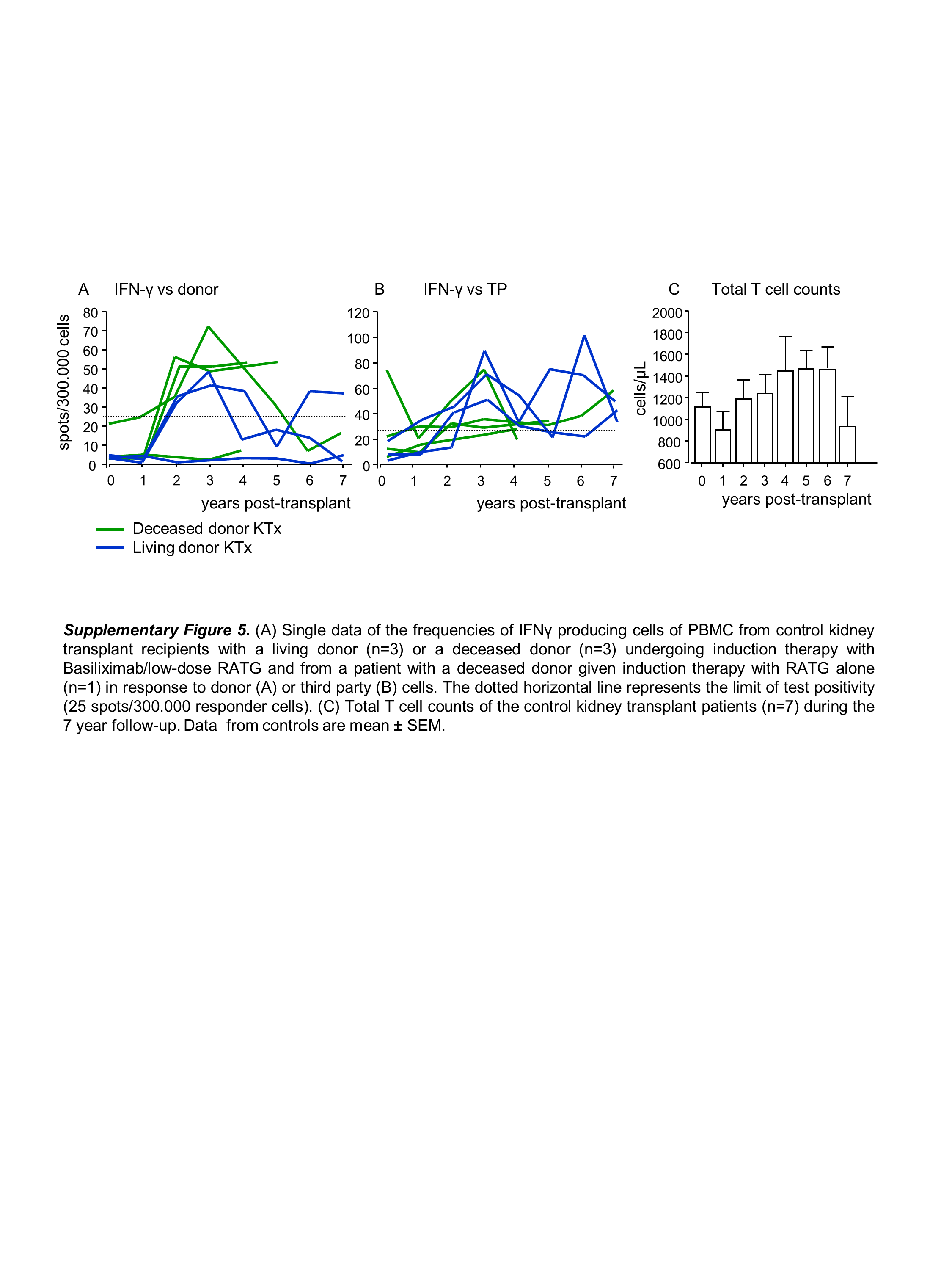

Supplement: Supplementary file 6 [file image_5.tif]
